# Supplementary material for: Carboxy-Methylation of the Catalytic Subunit of Protein Phosphatase 2A (PP2Ac) Integrates Methionine Availability with Methionine Addicted Cancer Cell Proliferation
Source: Biomolecules. 2025 Aug 22;15(9):1210. doi: 10.3390/biom15091210 (PMC12467028; doi:10.3390/biom15091210)
Supplement: Supplementary file 1 [file biomolecules-15-01210-s001.zip › Suppl. Figures.pdf]

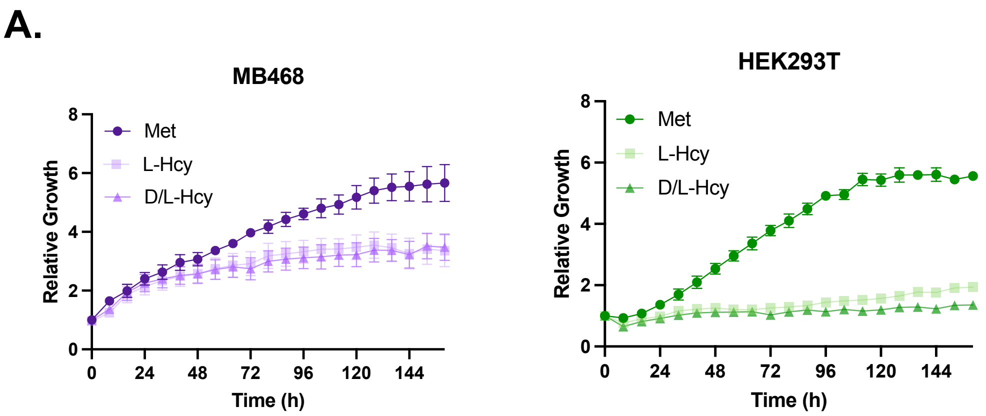

Figure S1: Comparison on cell growth in different homocysteine compounds
A) MB468 and HEK293T cells were treated with either 100 µM L-homocysteine or 370 µM DL-homocysteine and growth rates were compared over 6 days.

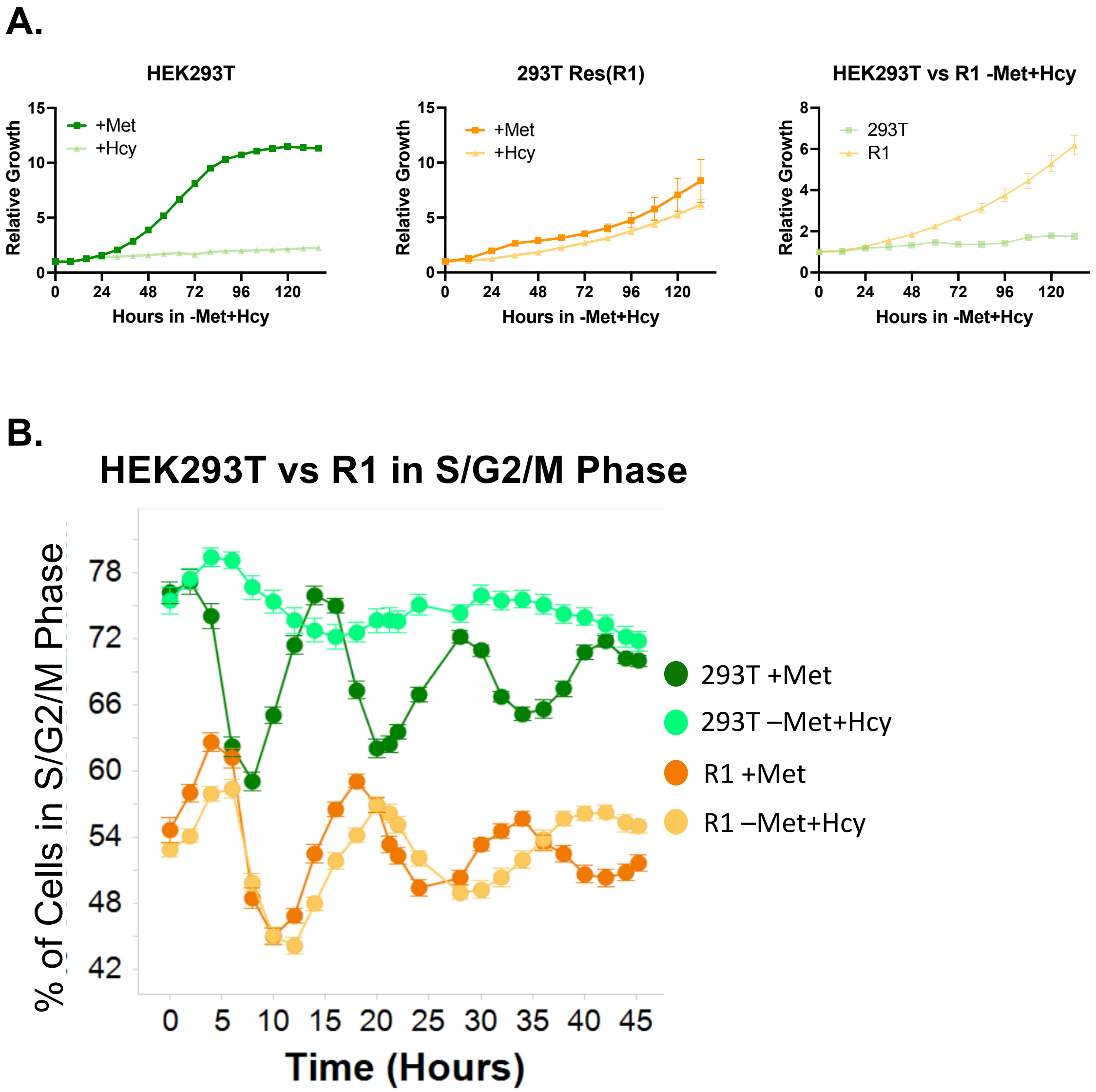

Figure S2: HEK293T and 293T Res1 (R1) cell growths comparison in different media

A) The methionine sensitive HEK293T and its resistant counterpart 293T Res1 (R1) were grown in either complete (+Met) media or methionine depleted (+Hcy) media and their proliferation was tracked. The HEK293T cells show a significant growth defect in -Met+Hcy media while the R1 cells have similar proliferation rates in both media types. B) Live-cell imaging was used to follow progression through the cell cycle after synchronization in early S-phase. R1 cells progressed normally in both complete and -Met+Hcy media. HEK293T cells could not progress in -Met+Hcy and arrested in S/G2/M.

A.

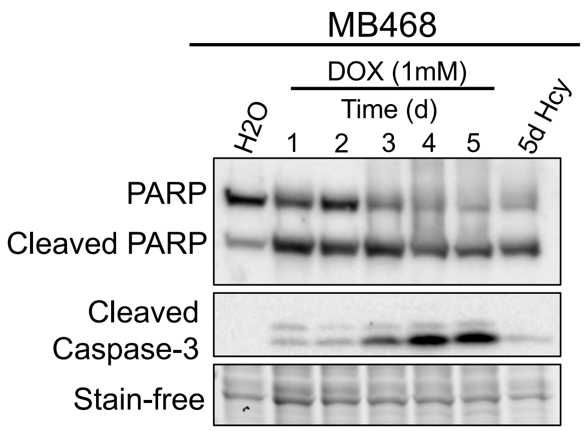

B.

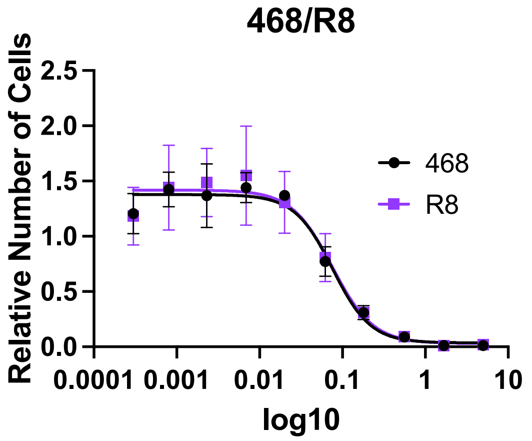

IC50 (μm)		SE
MB468	0.07591	0.01027
R8	0.07531	0.01770

Figure S3: Apoptosis controls for MB468/R8
A) 1mM doxorubicin (DOX) was used as a positive control for apoptosis and compared to 5 days growth in -Met+Hcy media. The positive control results in similar levels of PARP cleavage. B) IC_{50} for DOX was determined in both MB468 and R8 cells to compare intrinsic apoptotic potential.

A.

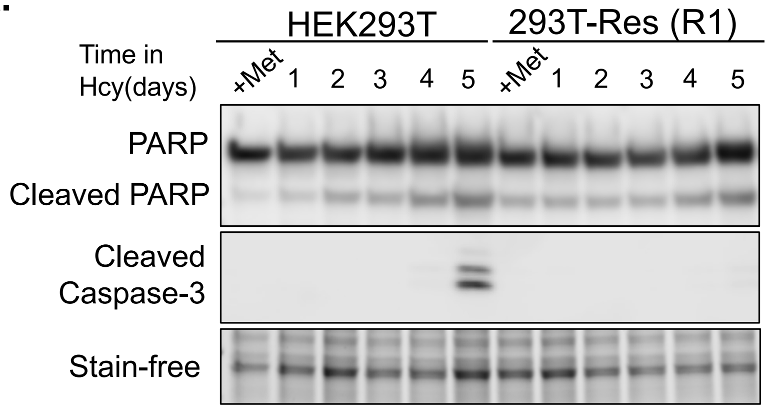

B.

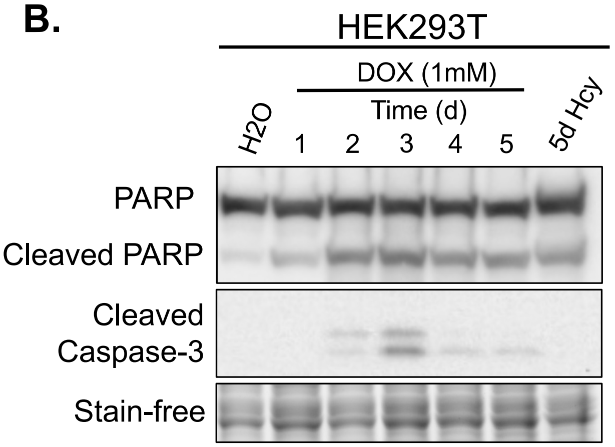

C.

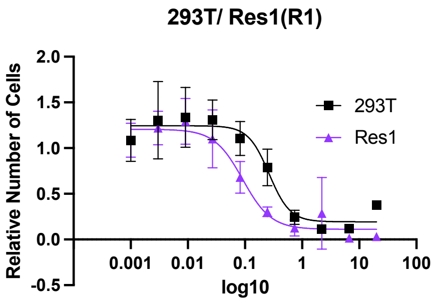

IC50 (μm)		SE
HEK293T	0.2639	0.05697
Res1	0.09091	0.02257

D.

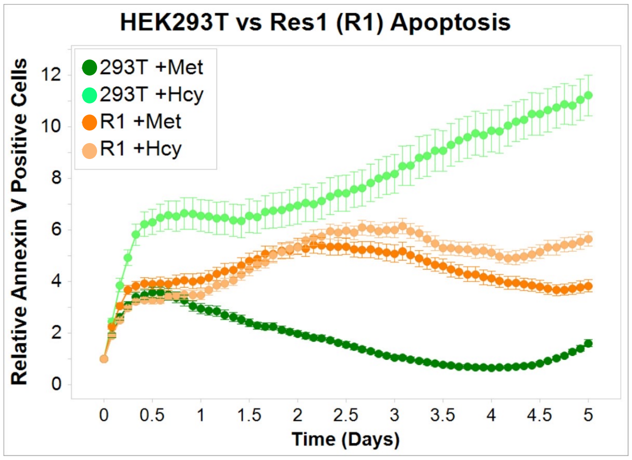

Figure S4: HEK293T cells have a modest increase in apoptosis when compared to R1 cells

A) Western blot was used to measure apoptosis by probing for PARP and Caspase-3 cleavage. B) 1mM Doxorubicin was used as a positive control. C) IC_{50} for doxorubicin (DOX) in both HEK293T and R1s was determined and compared. D) Live-cell imaging using Annexin V was used to measure apoptosis.

A.

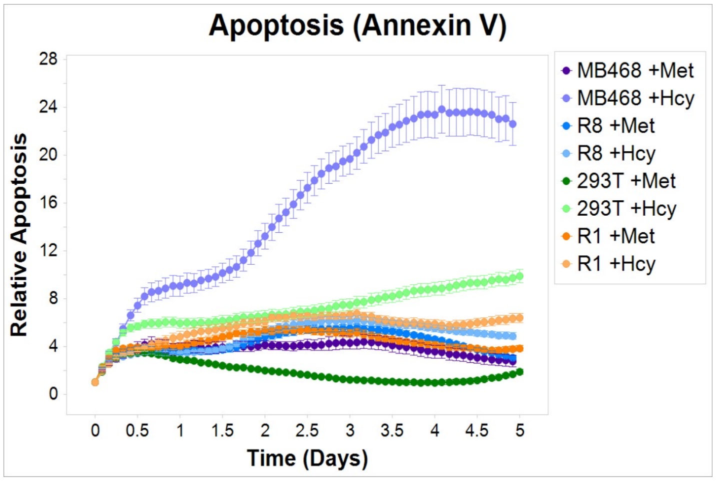
Apoptosis (Annexin V)
Relative Apoptosis
28
24
20
16
12
8
4
0
0
0.5
1
1.5
2
2.5
3
3.5
4
4.5
5
Time (Days)
MB468 +Met
MB468 +Hcy
R8 +Met
R8 +Hcy
293T +Met
293T +Hcy
R1 +Met
R1 +Hcy

B.

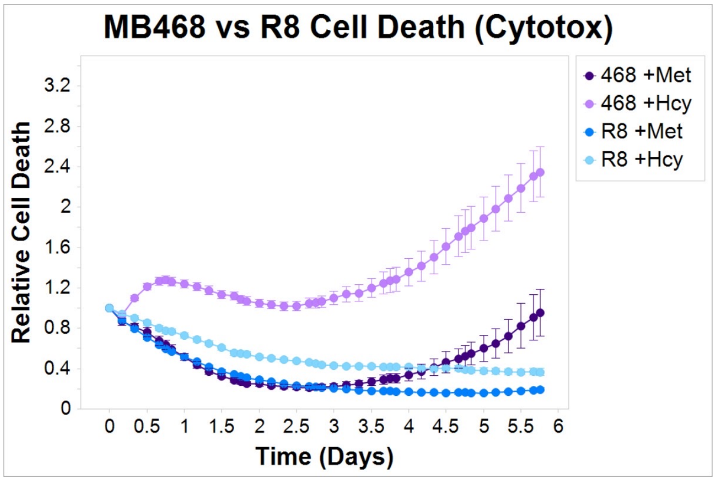
MB468 vs R8 Cell Death (Cytotox)
Relative Cell Death
3.2
2.8
2.4
2
1.6
1.2
0.8
0.4
0
0
0.5
1
1.5
2
2.5
3
3.5
4
4.5
5
5.5
6
Time (Days)
468 +Met
468 +Hcy
R8 +Met
R8 +Hcy

C.

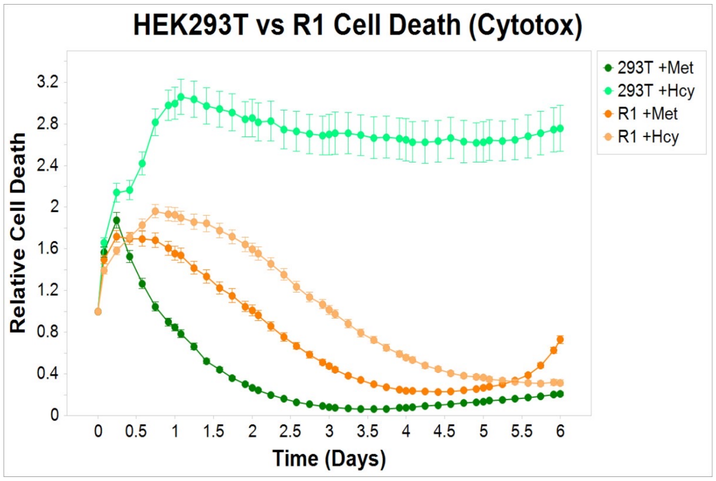
HEK293T vs R1 Cell Death (Cytotox)
Relative Cell Death
3.2
2.8
2.4
2
1.6
1.2
0.8
0.4
0
0
0.5
1
1.5
2
2.5
3
3.5
4
4.5
5
5.5
6
Time (Days)
293T +Met
293T +Hcy
R1 +Met
R1 +Hcy

Figure S5: HEK293T cells likely die through a non-apoptotic cell death pathway when cultured in -Met+Hcy media**.**
A) Apoptosis was measured using an Annexin V dye and live-cell imaging. B) General cell death was measured in live MB468 and R8 cells using cytotox. C) General cell death was measured in live HEK293T and R1 cells.

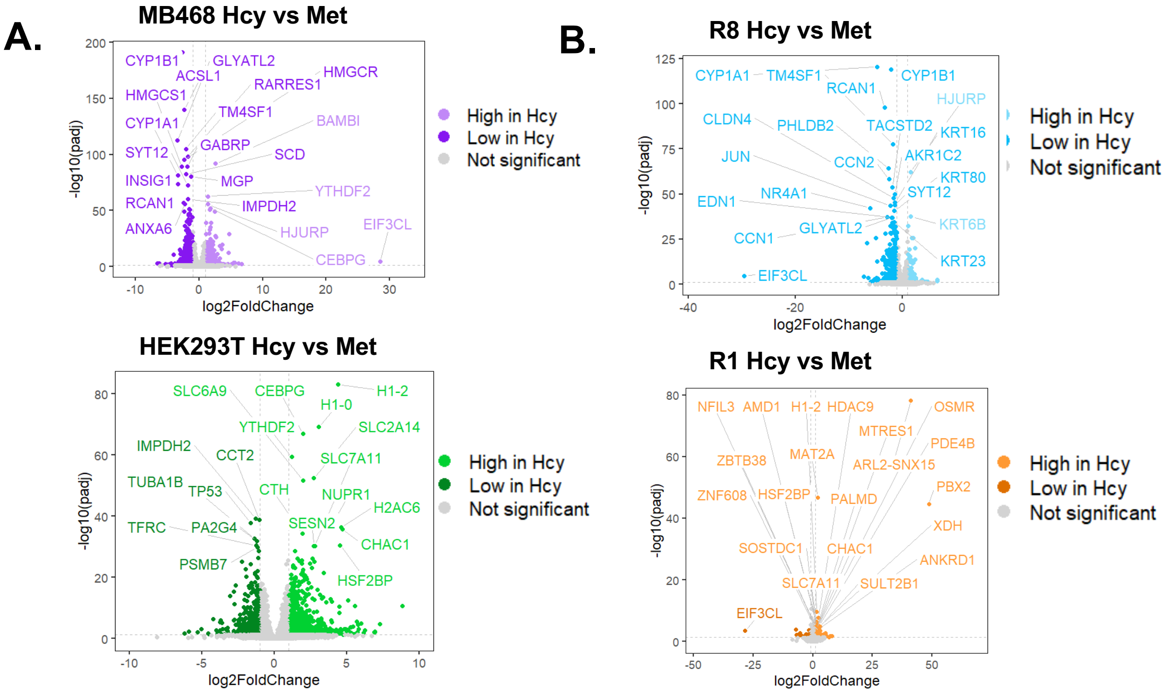

Figure S6: Volcano Plots

A) Transcriptional response for methionine sensitive cell lines. B) Transcriptional response for both methionine independent cell lines

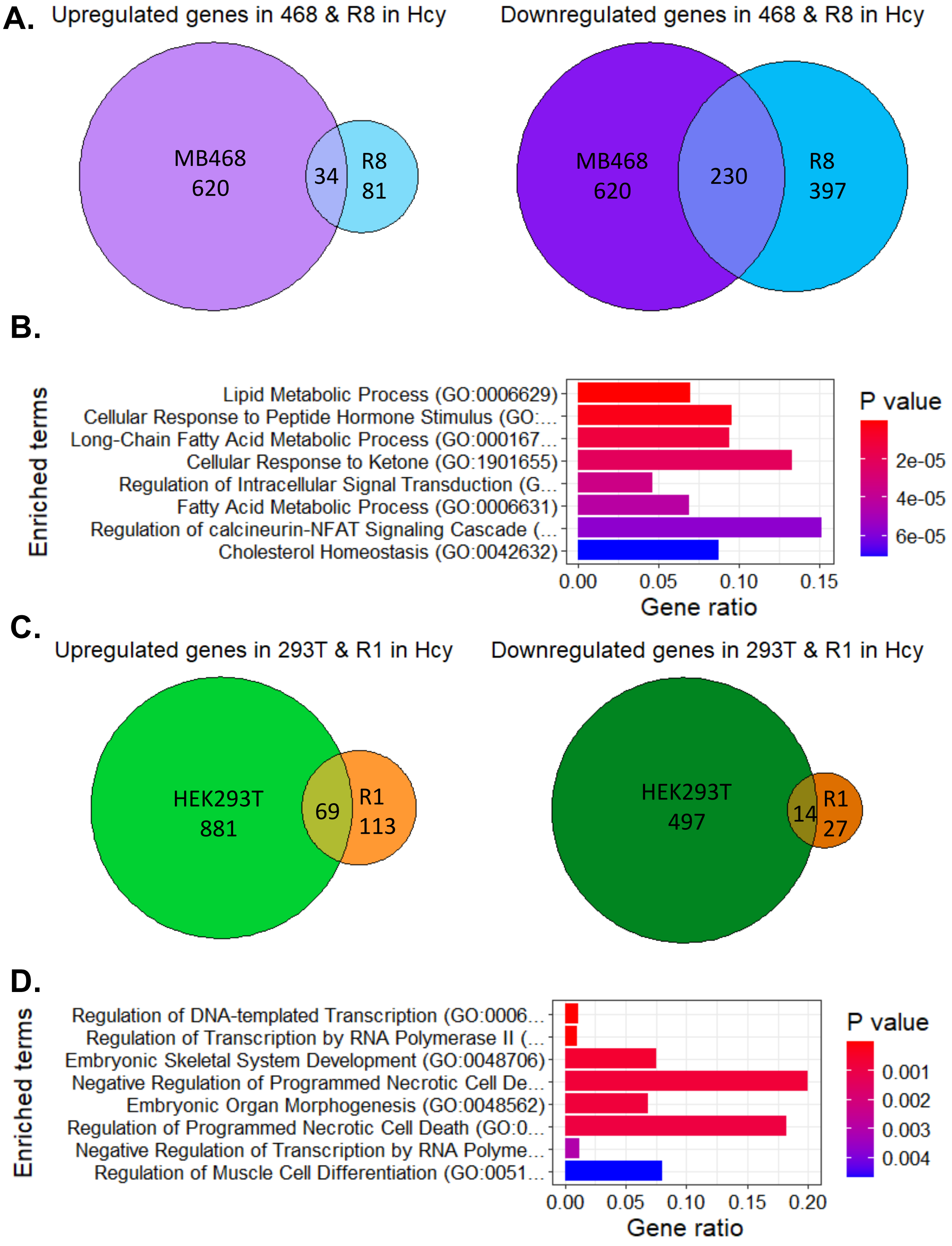
A.
Upregulated genes in 468 & R8 in Hcy
MB468
620
34
R8
81
Downregulated genes in 468 & R8 in Hcy
MB468
620
230
R8
397
B.
Enriched terms
Lipid Metabolic Process (GO:0006629)
Cellular Response to Peptide Hormone Stimulus (GO:...
Long-Chain Fatty Acid Metabolic Process (GO:000167...
Cellular Response to Ketone (GO:1901655)
Regulation of Intracellular Signal Transduction (G...
Fatty Acid Metabolic Process (GO:0006631)
Regulation of calcineurin-NFAT Signaling Cascade (...
Cholesterol Homeostasis (GO:0042632)
0.00
0.05
0.10
0.15
Gene ratio
P value
2e-05
4e-05
6e-05
C.
Upregulated genes in 293T & R1 in Hcy
HEK293T
881
69
R1
113
Downregulated genes in 293T & R1 in Hcy
HEK293T
497
14
R1
27
D.
Enriched terms
Regulation of DNA-templated Transcription (GO:0006...
Regulation of Transcription by RNA Polymerase II (...
Embryonic Skeletal System Development (GO:0048706)
Negative Regulation of Programmed Necrotic Cell De...
Embryonic Organ Morphogenesis (GO:0048562)
Regulation of Programmed Necrotic Cell Death (GO:0...
Negative Regulation of Transcription by RNA Polyme...
Regulation of Muscle Cell Differentiation (GO:0051...
0.00
0.05
0.10
0.15
0.20
Gene ratio
P value
0.001
0.002
0.003
0.004

Figure S7: Comparisons between parental and revertant cell lines
A) Genes that were upregulated and downregulated in MB468 and R8 cells in response to growth in -Met+Hcy media. B) GO term analysis for the overlapping transcriptional response between MB468 and R8 cells. C) Genes that were upregulated and downregulated in HEK293T and R1 cells in response to growth in -Met+Hcy media. D) GO term analysis for the overlapping transcriptional response between HEK293T and R1 cells.

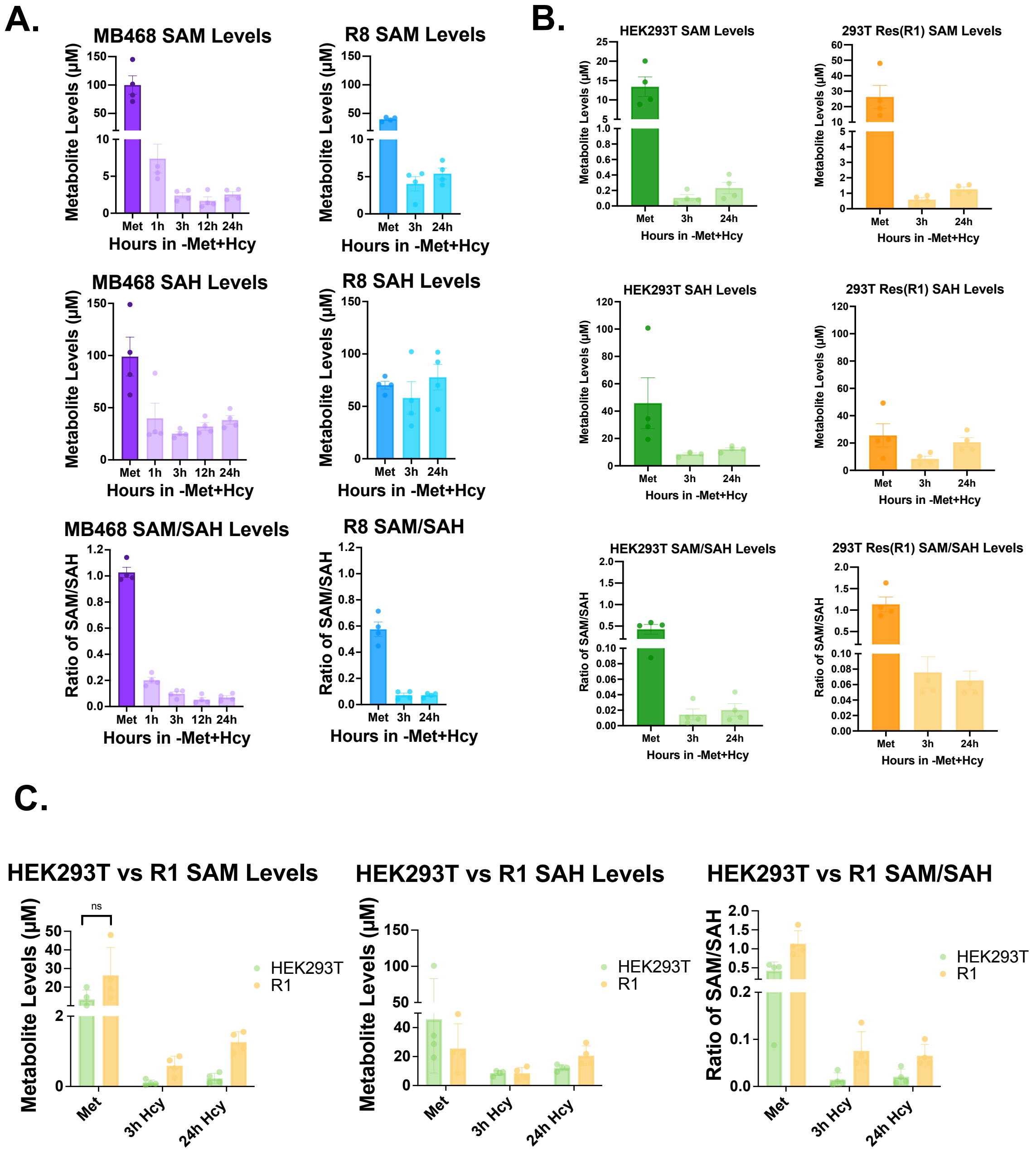

Figure S8: Metabolomics

A) Figure 3A represented as individual cell lines. B) Experiment was performed as Fig 3A but using HEK293T and R1 cells. The methylation potential was calculated using the SAM/SAH ratio. C) Data from S5B displayed as cell groupings for better comparison.

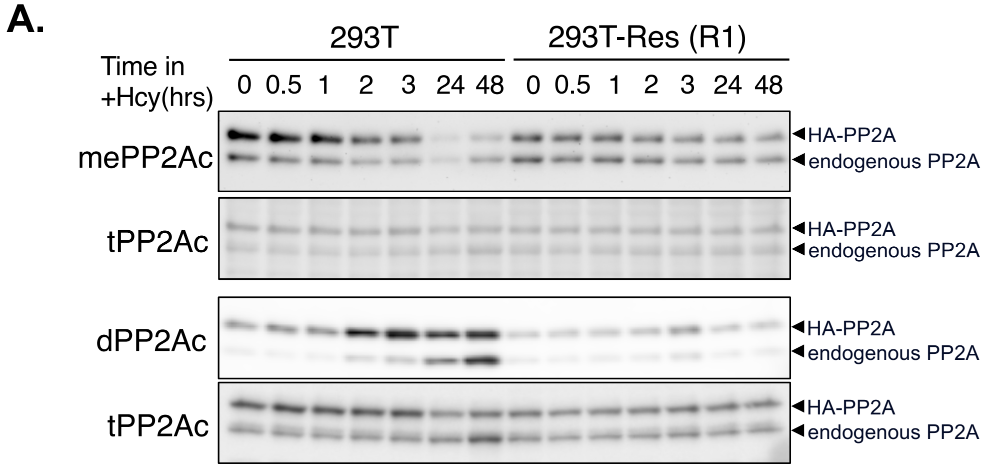

Figure S9: PP2Ac methylation responds in HEK293T but not R1 cells to growth in -Met/+Hcy media.

A) HEK293T and R1 cells were grown in -Met/+Hcy media for up to 3 days, with samples being collected at different time points. Whole cell lysates were then analyzed for PP2Ac methylation.

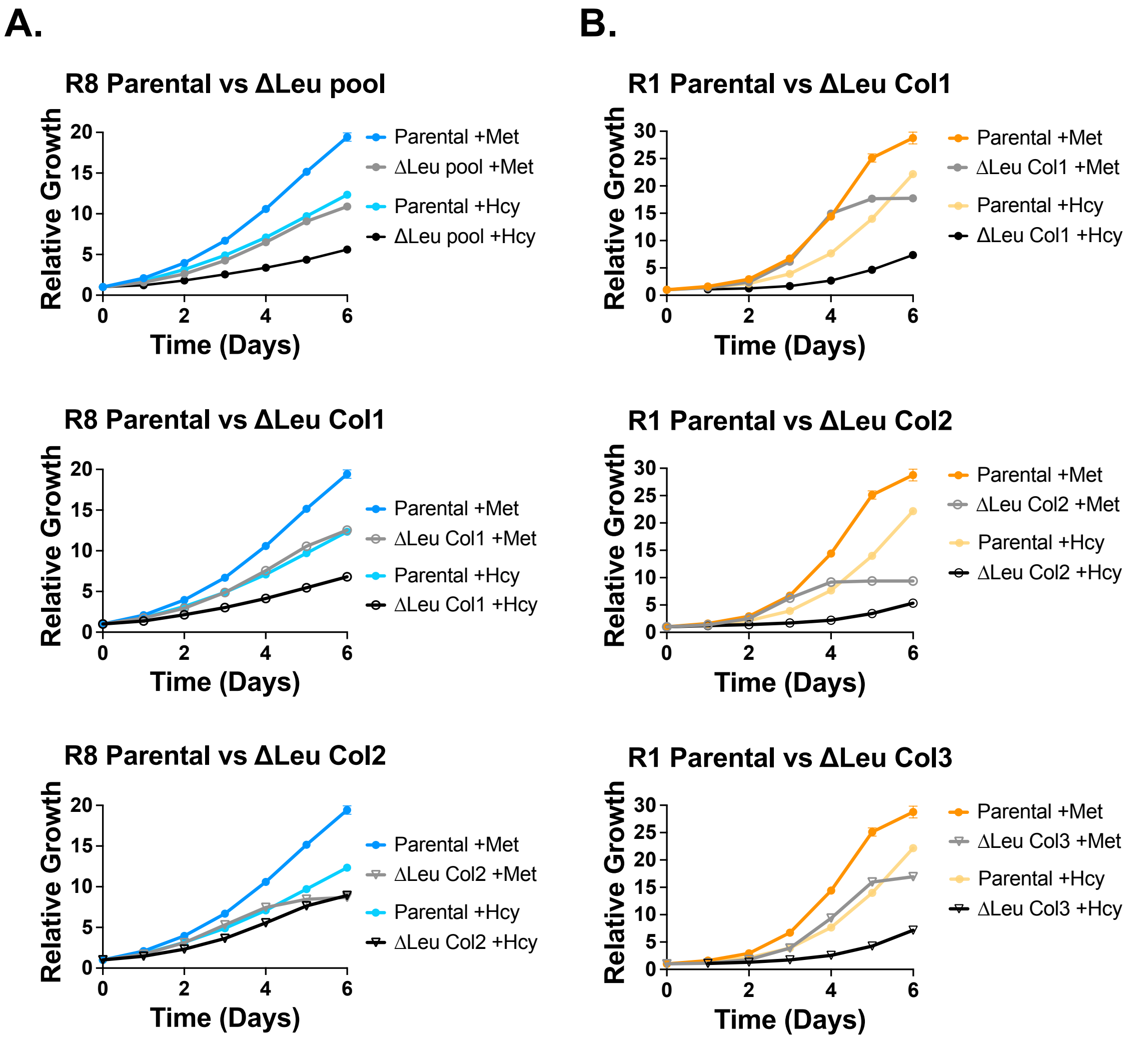

Figure S10: ΔLeu309 slows down, but does not inhibit cell proliferation in complete media

A) R8 cells cultured in +Met or -Met+Hcy compared to the ΔLeu309 pool or individual colonies. B) R1 cells cultured in +Met or -Met+Hcy compared to each individual ΔLeu309 colony

A.

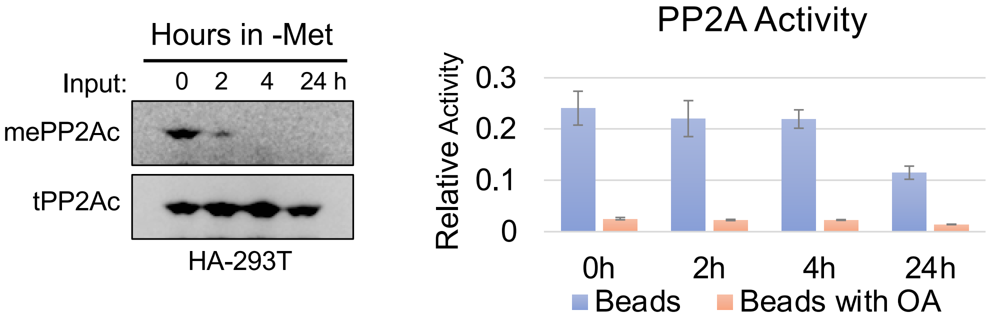

Figure S11: Reduced PP2Ac methylation in response to methionine depletion does not affect intrinsic PP2A phosphatase activity.

A) HEK293T cells with an N-terminal tagged PP2Ac was immunopurified and activity of the phosphatase was analyzed. The PP2A inhibitor okadaic acid was used as a control. While methylation of PP2Ac is lost as early as 2 hours in -Met media, intrinsic phosphatase activity is unaffected.
