# Supplementary material for: Carboxy-Methylation of the Catalytic Subunit of Protein Phosphatase 2A (PP2Ac) Integrates Methionine Availability with Methionine Addicted Cancer Cell Proliferation
Source: Biomolecules. 2025 Aug 22;15(9):1210. doi: 10.3390/biom15091210 (PMC12467028; doi:10.3390/biom15091210)
Supplement: Supplementary file 1 [file biomolecules-15-01210-s001.zip › Uncropped blots_updated.pdf]

A.

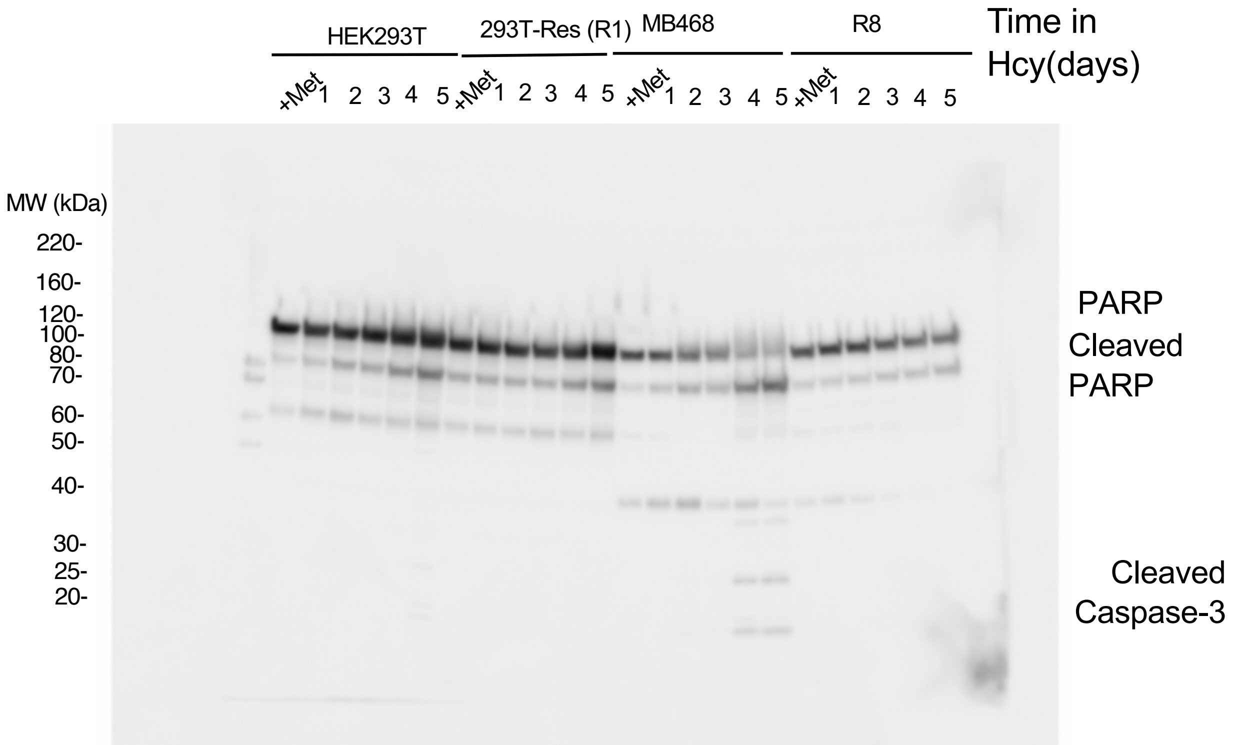

B.

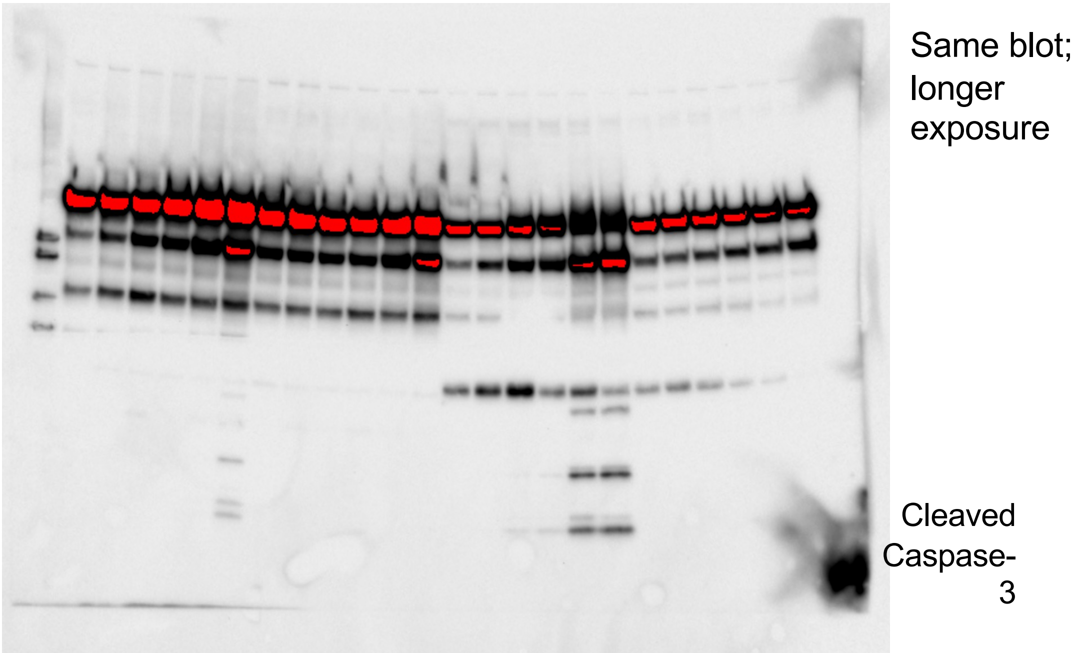

C.

MB468 / R8

	Met	1	2	3	4	5	Met	1	2	3	4	5
PARP	2338341	2444584	2364310	2136264	1567125	1107266	2707411	2910816	3041324	2436700	2396763	2418484
Cleaved PARP	199985	449326	730496	744822	1694475	2251838	375666	289992	365820	408650	459012	685299

HEK293T / R1

	Met	1	2	3	4	5	Met	1	2	3	4	5
PARP	4827282	4423399	4647482	5564027	6127700	5623008	4952480	5025692	3978724	4352610	4538170	5769848
Cleaved PARP	185687	310532	700379	808404	1360361	1437768	640328	637140	529759	660243	1033500	1549688

D.

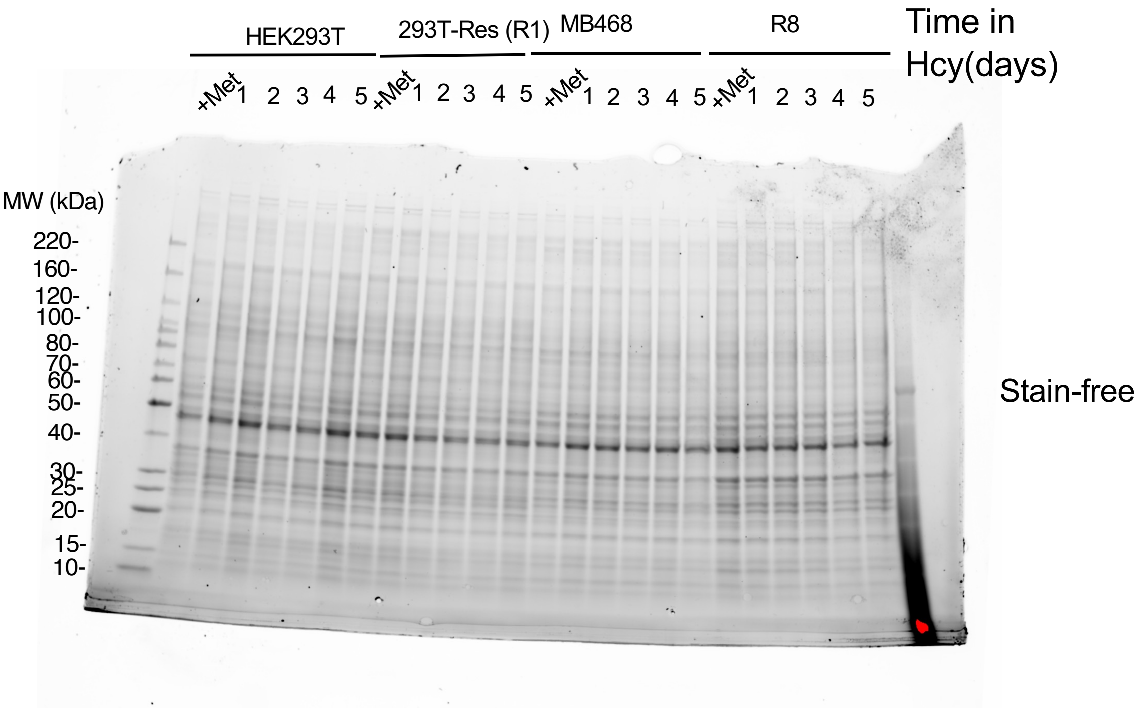

E.

Lane No.	Adj. Total Band Vol. (Int)
1	43441706
2	63789110
3	75969278
4	51048305
5	52418597
6	78807960
7	64552642
8	74194351
9	55643742
10	46072180
11	46056626
12	42528486
13	56677698
14	64282295
15	56583989
16	60409734
17	60844861
18	42830711
19	72750293
20	71915074
21	68174106
22	64034201
23	48406050
24	65993081

Figure S12: Uncropped blots relating to Figures 1E and S4A

A) Shorter exposure B) Longer exposure C) Densitometry (using total adjusted band volume from BioRad ImageLab) D) Stain-free loading control E) Densitometry (using total adjusted band volume from BioRad ImageLab) for stain-free control

A.

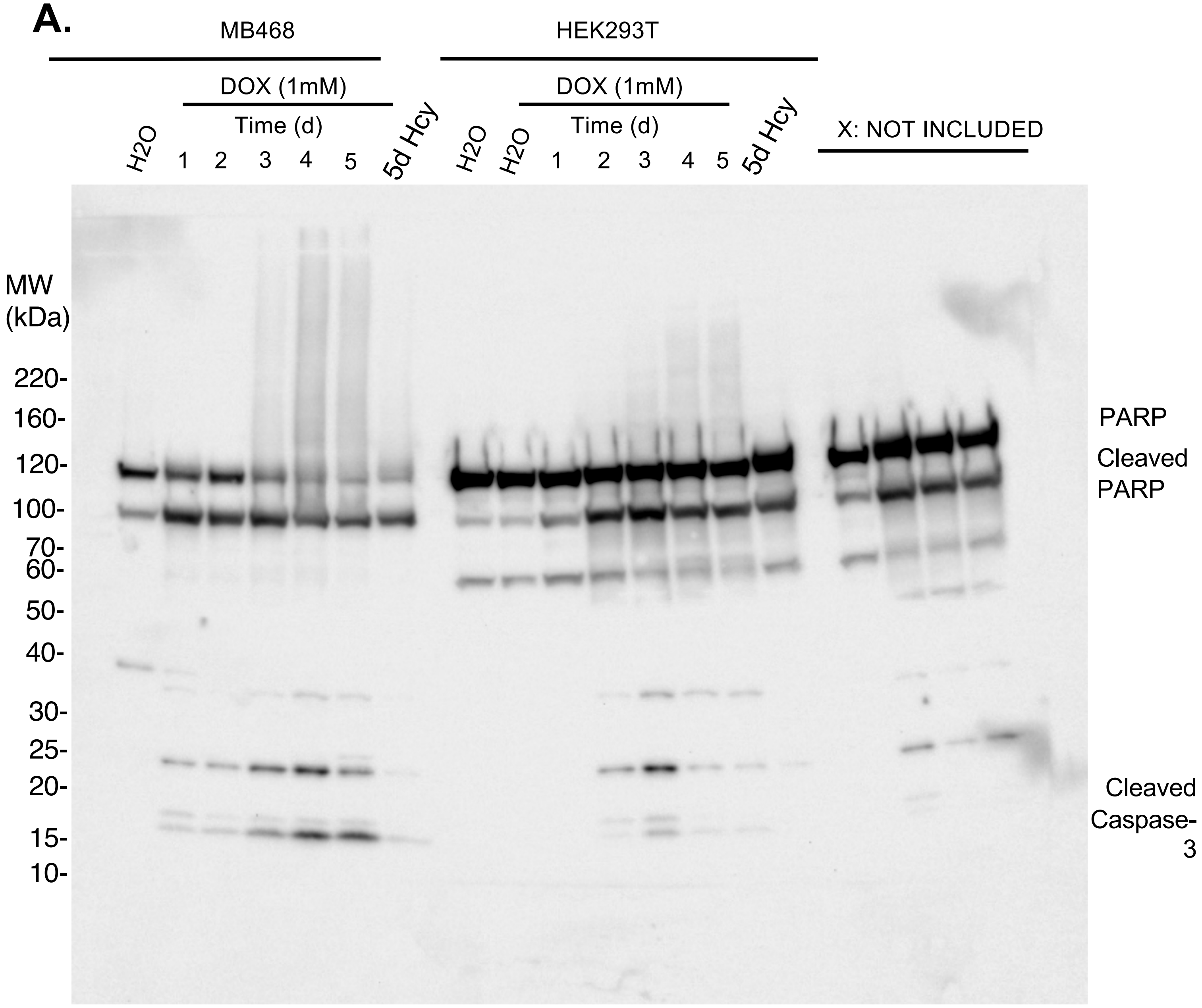

B.

MB468 HEK293T

	H20	1	2	3	4	5	5d Hcy	H20	H20	1	2	3	4	5	5d Hcy
PARP	1636736	1331484	1553414	808780	366966	307528	112768	5244704	3525184	4000416	3279120	3101200	3412920	3342898	3862912
Cleaved PARP	612000	1487808	1204718	1146264	835142	742664	1023296	185504	177346	532576	1216488	1411600	1249388	1227070	1386240

C.

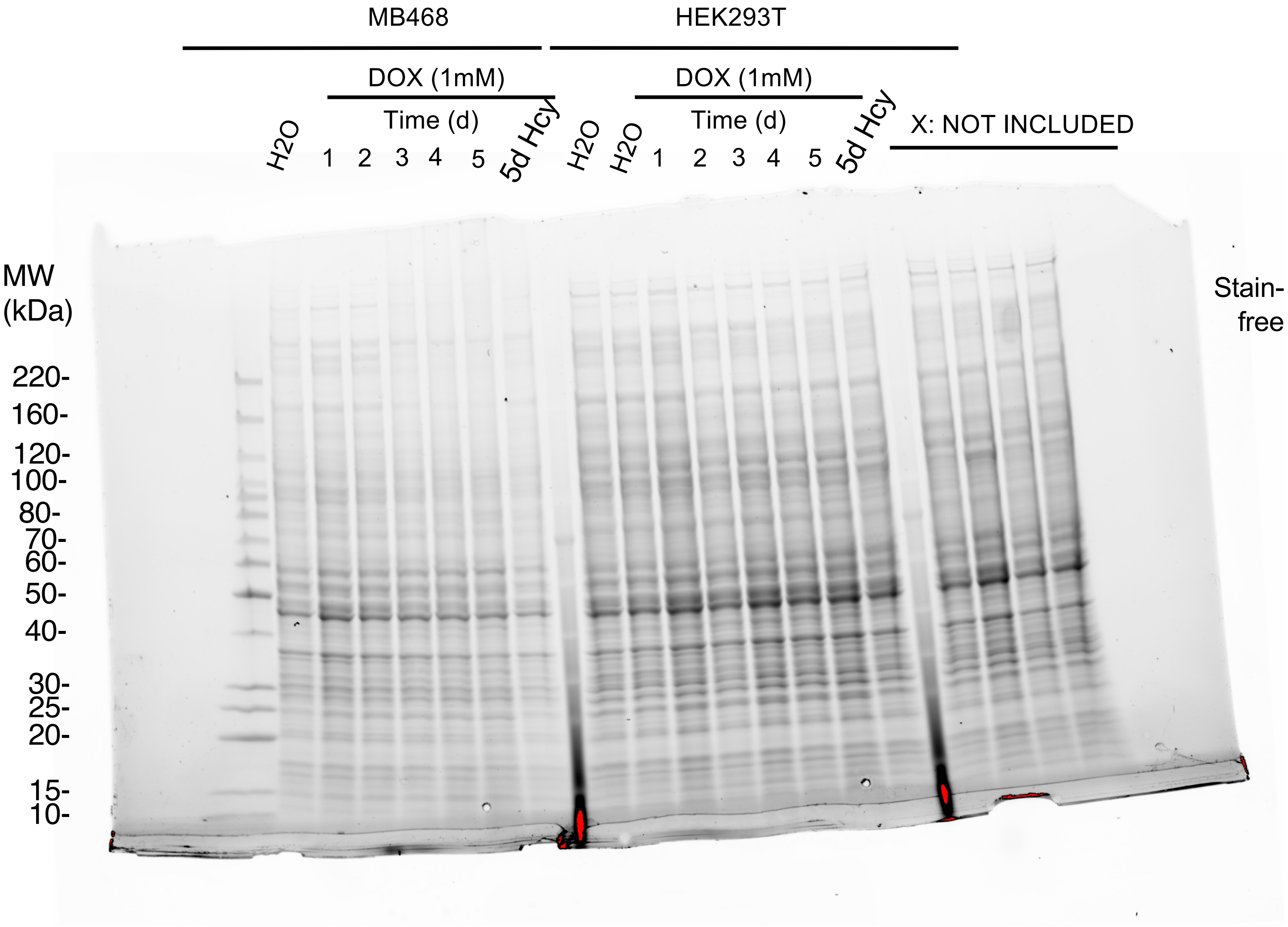

D.

Lane No.	Adj. Total Band Vol. (Int)
1	54172158
2	76492552
3	63071874
4	54745030
5	49306180
6	42492518
7	32804194
8	86589421
9	94902024
10	117108490
11	94448534
12	117277867
13	101184325
14	127288381
15	77683039

Figure S13: Uncropped blots relating to Figures S3A and S4B

A) Uncropped Blot B) Densitometry (using total adjusted band volume from BioRad ImageLab) C) Stain-free loading control D) Densitometry (using total adjusted band volume from BioRad ImageLab) for stain-free control

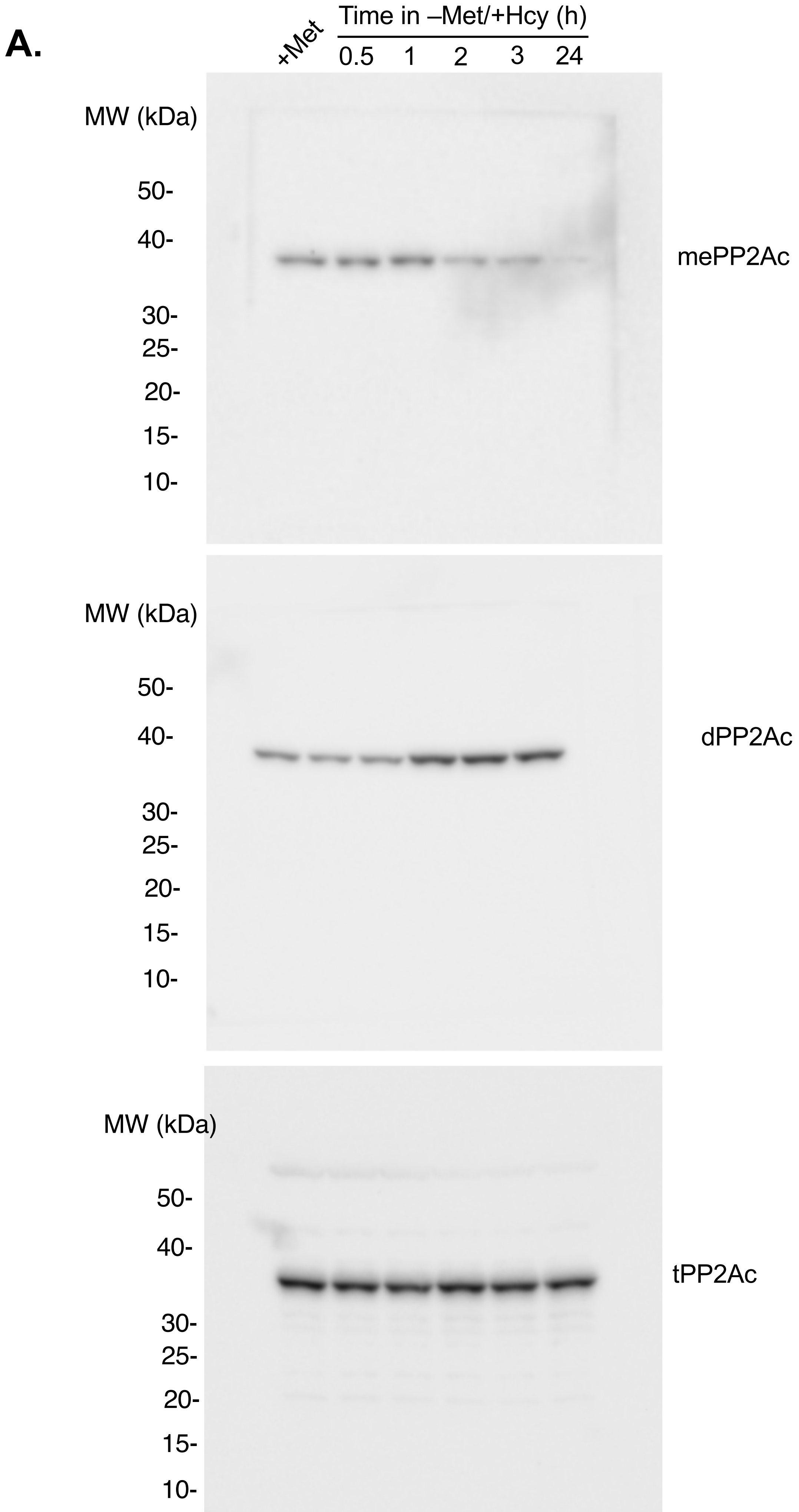
A.
+Met
Time in –Met/+Hcy (h)
0.5
1
2
3
24
MW (kDa)
50-
40-
30-
25-
20-
15-
10-
mePP2Ac
MW (kDa)
50-
40-
30-
25-
20-
15-
10-
dPP2Ac
MW (kDa)
50-
40-
30-
25-
20-
15-
10-
tPP2Ac

B.

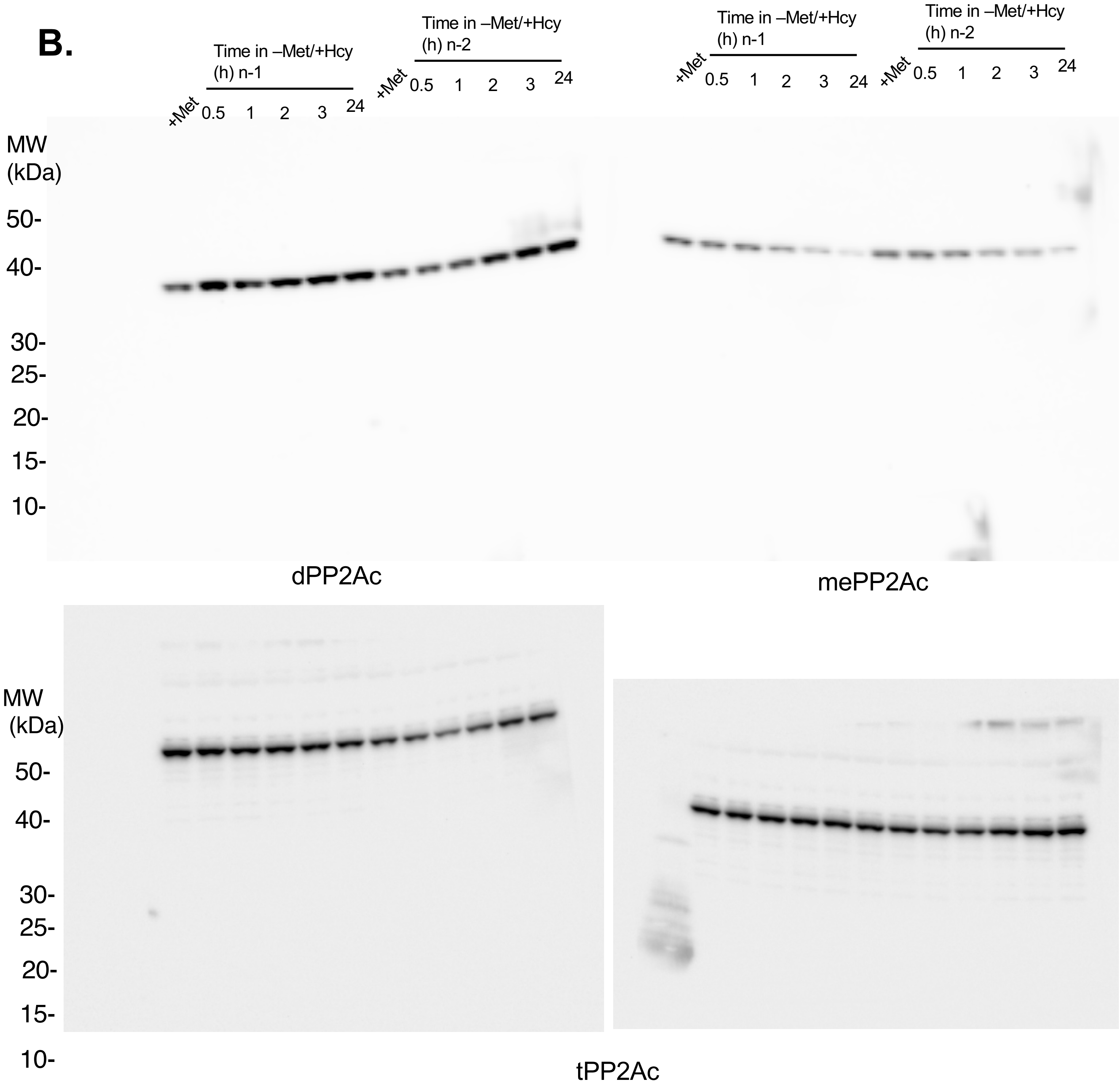

Replicates used for quantification

C.

		dPP2Ac	tPP2Ac	mPP2Ac	tPP2Ac
N-1	Met	1503909	2968280	886686	1820595
	Hcy 0.5h	1197735	2674595	607784	1783355
	Hcy 1h	1408803	2430050	665210	2138010
	Hcy 2h	2761836	2854845	209440	2563330
	Hcy 3h	3040323	2565185	198730	2966215
	Hcy 24h	2954259	2525740	43962	3012765
N-2	Met	2069808	8679783	1328886	6613200
	Hcy 0.5h	3954846	7817766	1293474	6360660
	Hcy 1h	2936888	7037093	1243268	6404160
	Hcy 2h	3582080	7250001	915798	6472500
	Hcy 3h	3918090	6665155	576810	6553740
	Hcy 24h	3791730	5674798	329914	6366090
N-3	Met	1988506	4876920	1447446	5761800
	Hcy 0.5h	1847274	3741979	1387412	5121780
	Hcy 1h	2026362	2722723	1221090	4718490
	Hcy 2h	2889027	3776360	842764	5755770
	Hcy 3h	2998658	5066096	787254	7203456
	Hcy 24h	3473440	4612304	464204	6787350

Figure S14 : Uncropped blots relating to Figure 3C
A and B) Uncropped blots C) Densitometry (using total adjusted band volume from BioRad ImageLab)
Note: Membranes were cut to accommodate multiple antibodies

A.

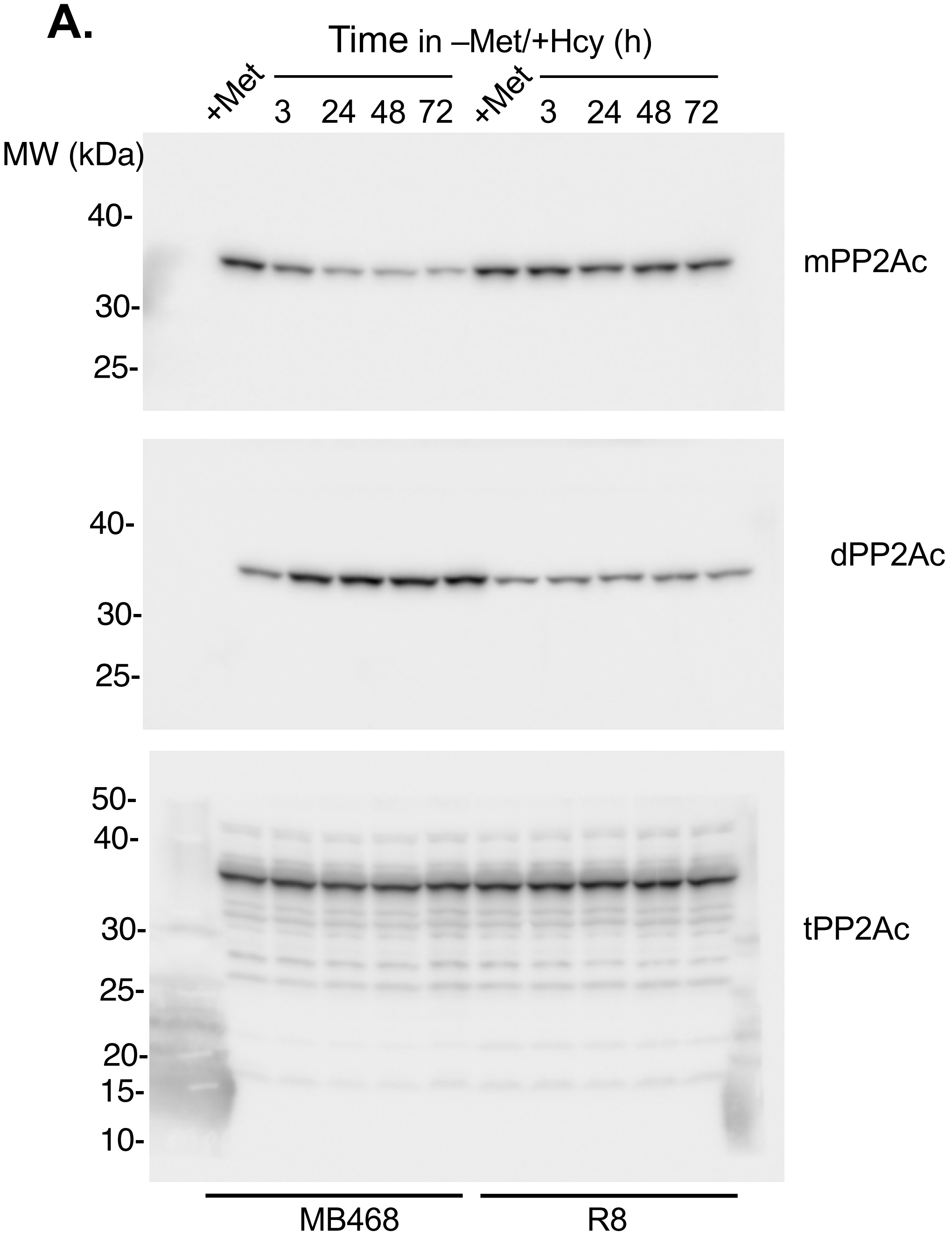

B.

	MB468					R8				
	**Met**	**3**	**24**	**48**	**72**	**Met**	**3**	**24**	**48**	**72**
**mPP2A**	2602576	1498488	764788	675874	639102	2433622	2482226	1994823	2396183	2196256
**dPP2A**	1046472	2252572	2483768	2508576	2421524	834932	904652	968032	1097568	811239
**tPP2A**	42746352	43911384	38988312	48473436	43090872	52507224	54568272	50415816	50323812	35084016

Figure S15: Uncropped blots relating to Figure 3E
A) Uncropped blot B) Densitometry (using total adjusted band volume from BioRad ImageLab)
Note: Membranes were cut to accommodate multiple antibodies

A.

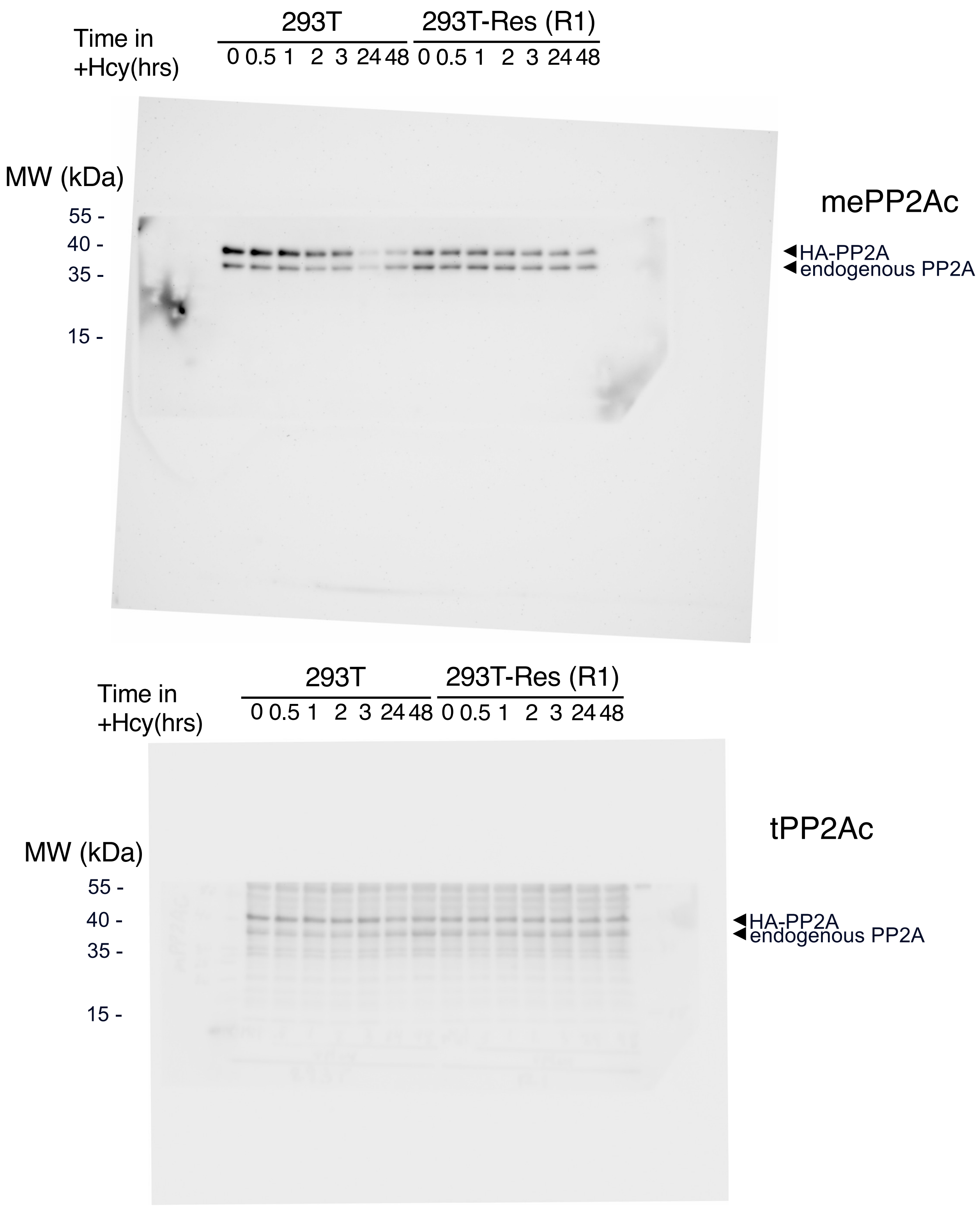
Time in +Hcy(hrs)
293T
293T-Res (R1)
0 0.5 1 2 3 24 48 0 0.5 1 2 3 24 48
MW (kDa)
55 -
40 -
35 -
15 -
mePP2Ac
HA-PP2A
endogenous PP2A
Time in +Hcy(hrs)
293T
293T-Res (R1)
0 0.5 1 2 3 24 48 0 0.5 1 2 3 24 48
MW (kDa)
55 -
40 -
35 -
15 -
tPP2Ac
HA-PP2A
endogenous PP2A

B.

		Lane	Band No.	**Methyl PP2AC**	**Total PP2AC**
293T	0	1	1	5735891	7299936
		1	2	2482879	3427200
	0.5	2	1	4998000	6468224
		2	2	1801534	2778384
	1	3	1	4658381	6423648
		3	2	1839166	2672320
	2	4	1	2921625	5808208
		4	2	1061291	2527952
	3	5	1	2535260	6604640
		5	2	1103921	3110128
	24	6	1	374262	3779888
		6	2	509208	3916864
	48	7	1	859264	5128816
		7	2	1497342	5762624
293T-Res (R1)	0	8	1	2849546	4331488
		8	2	2672852	3982048
	0.5	9	1	2502871	4348736
		9	2	2009980	2896544
	1	10	1	2654722	4388608
		10	2	2312555	3307920
	2	11	1	2192554	4515056
		11	2	2102590	3637536
	3	12	1	1867145	4721024
		12	2	1681974	3628240
	24	13	1	1732836	4400816
		13	2	1726074	4060224
	48	14	1	1500135	4872112
		14	2	1562757	4083408

C.

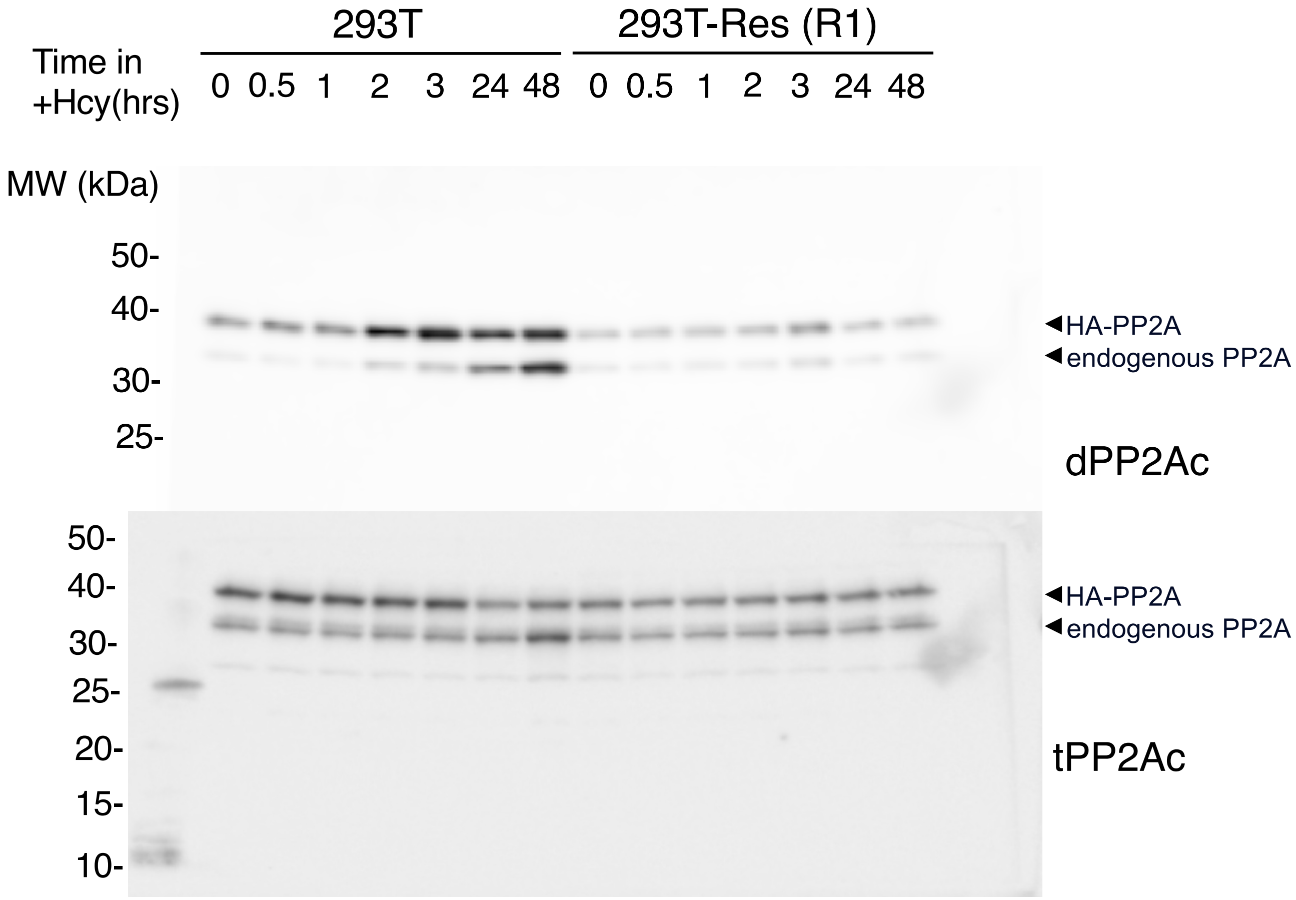

293T
293T-Res (R1)
Time in +Hcy(hrs)
0 0.5 1 2 3 24 48 0 0.5 1 2 3 24 48
MW (kDa)
50-
40-
30-
25-
HA-PP2A
endogenous PP2A
dPP2Ac
50-
40-
30-
25-
20-
15-
10-
HA-PP2A
endogenous PP2A
tPP2Ac

C.

	293T							293T-Res (R1)						
	**Met**	**0.5**	**1**	**2**	**3**	**24**	**48**	**Met**	**0.5**	**1**	**2**	**3**	**24**	**48**
**mPP2A**	831996	1622052	838656	522378	282240	20520	103086	533844	555228	1011456	671094	460206	613800	1383048
**tPP2A**	1747818	1938168	1872448	1652319	2216484	1639089	2168829	1712610	1787427	2086938	1816803	1806192	1487781	1472175
**dPP2A**	882854	990066	820000	1808268	2407002	2149590	3482472	552662	552812	615030	710892	1007281	555009	509404
**tPP2A**	2278233	2530548	2358288	2409534	2464479	1792449	2651940	1937358	1425978	1631475	1722951	1971216	1796904	1665252

Figure S16 : Uncropped blots relating to Figure S9

A) Uncropped blots for mePP2A B) Densitometry (using total adjusted band volume from BioRad ImageLab) C) Uncropped blots for dPP2A B) Densitometry

Note: Membranes were cut to accommodate multiple antibodies

A.

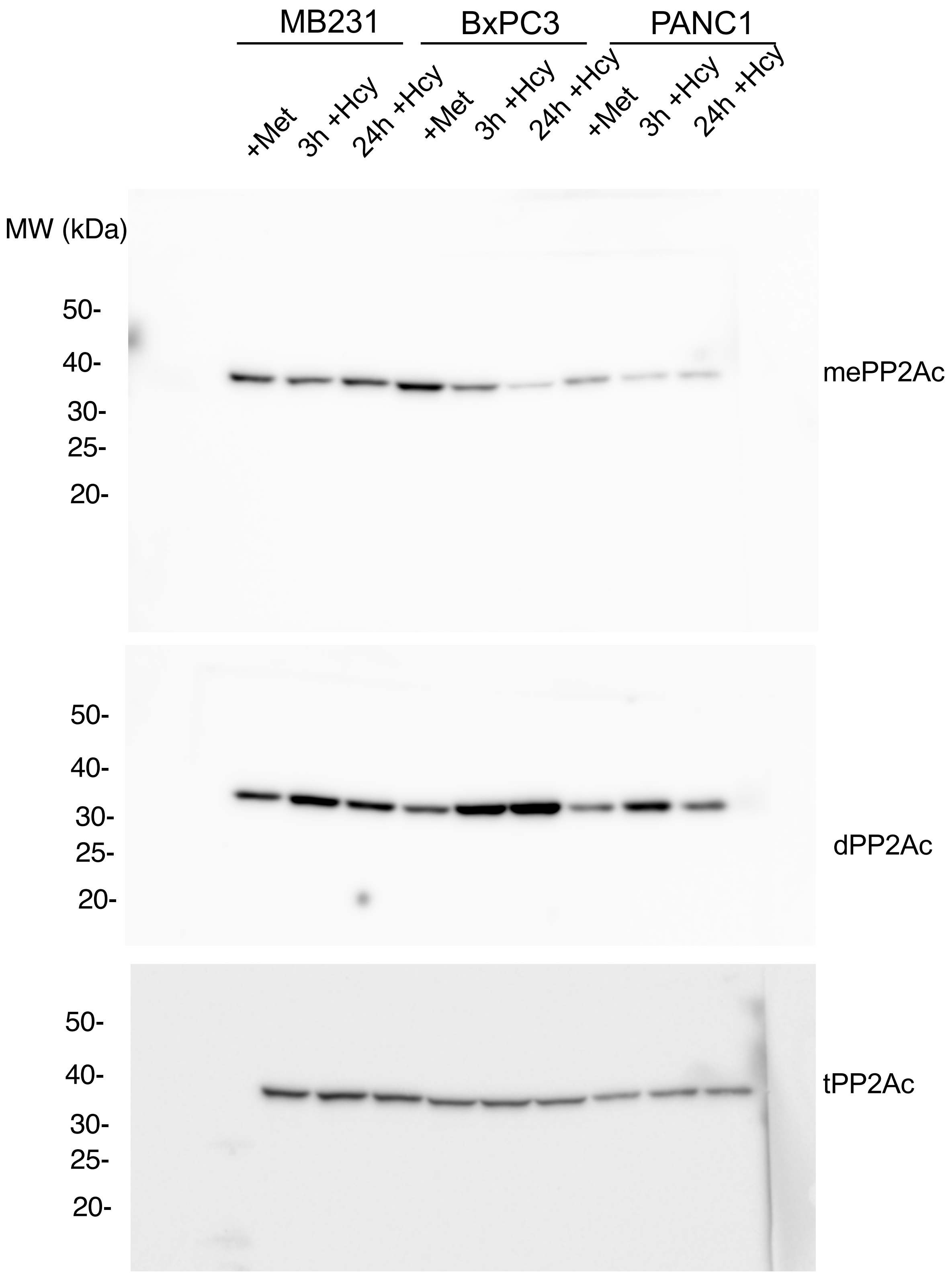

MB231
BxPC3
PANC1
+Met
3h +Hcy
24h +Hcy
+Met
3h +Hcy
24h +Hcy
+Met
3h +Hcy
24h +Hcy
MW (kDa)
50-
40-
30-
25-
20-
mePP2Ac
50-
40-
30-
25-
20-
dPP2Ac
50-
40-
30-
25-
20-
tPP2Ac

B.

	MB231			BxPC3			PANC1		
	**Met**	**3**	**24**	**Met**	**3**	**24**	**Met**	**3**	**24**
**mPP2A**	589472	452660	563004	773656	367874	100282	239356	101400	92326
**dPP2A**	1139320	1874574	1364766	986856	2209922	2695524	785798	1469104	846066
**tPP2A**	10959339	11780784	10107258	7949076	8147865	6843006	4511151	5799381	5892117

Figure S17 : Uncropped blots relating to Figure 4

A) Uncropped blots B) Densitometry (using total adjusted band volume from BioRad ImageLab)

Note: Membranes were cut to accommodate multiple antibodies

A.

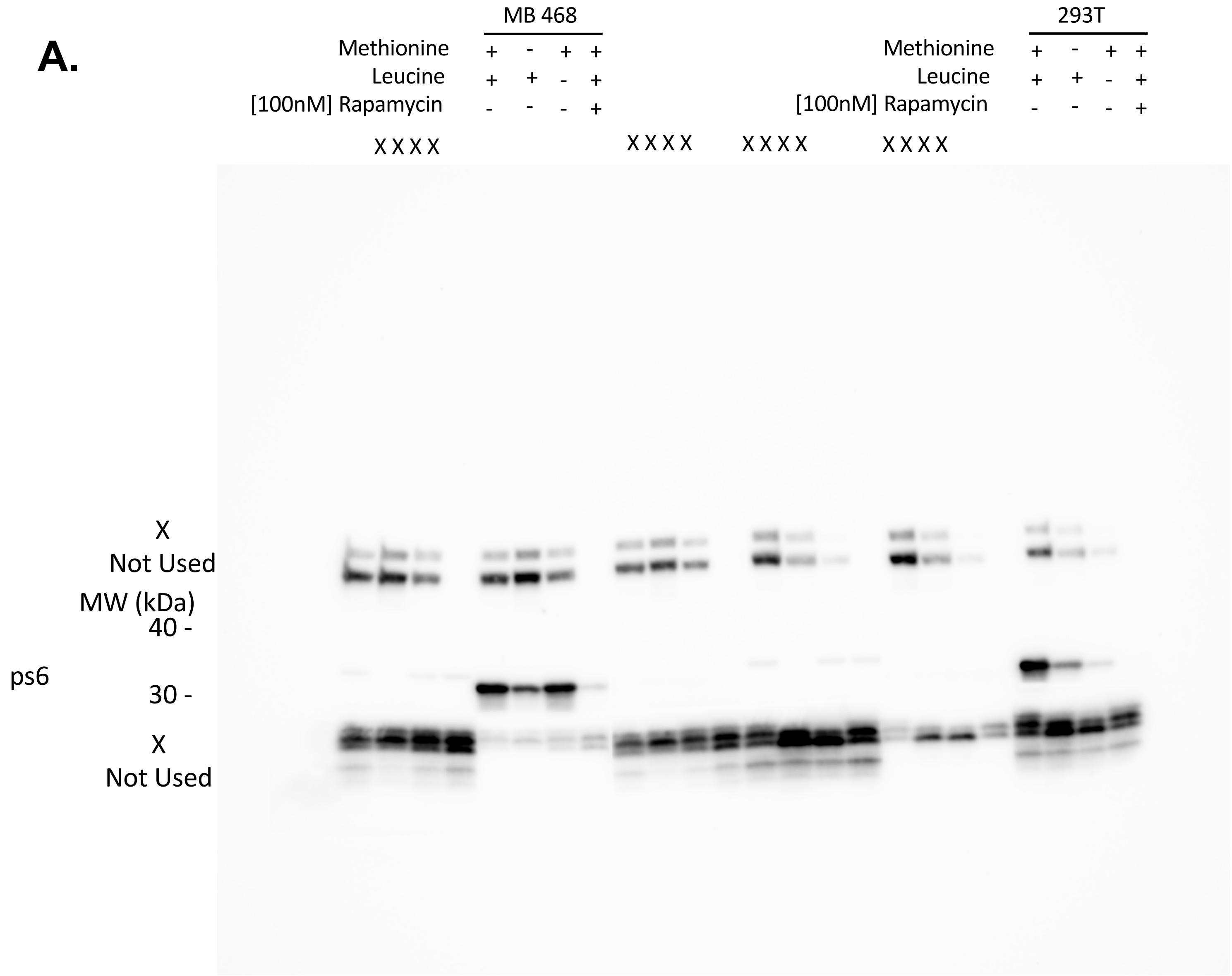
MB 468
293T
Methionine + - + +
Leucine + + - +
[100nM] Rapamycin - - - +
Methionine + - + +
Leucine + + - +
[100nM] Rapamycin - - - +
XXXX
XXXX
XXXX
XXXX
X
Not Used
MW (kDa)
40 -
ps6
30 -
X
Not Used

B.

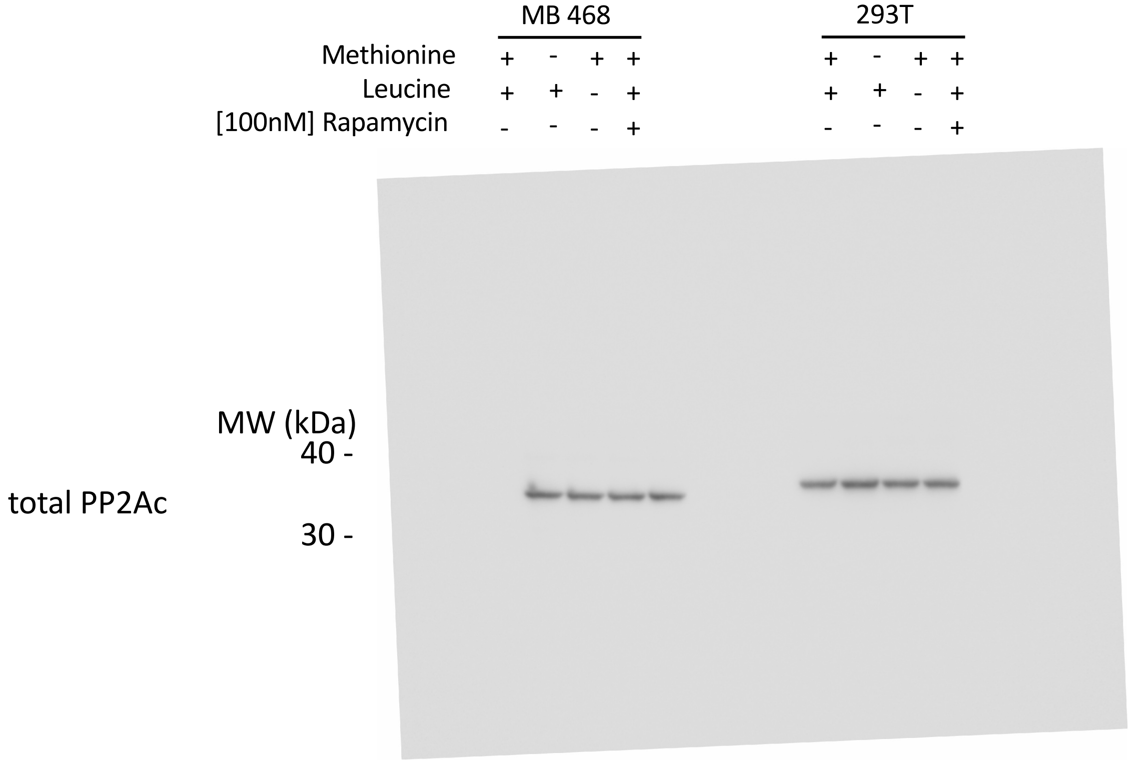
MB 468
293T
Methionine + - + +
Leucine + + - +
[100nM] Rapamycin - - - +
Methionine + - + +
Leucine + + - +
[100nM] Rapamycin - - - +
MW (kDa)
40 -
total PP2Ac
30 -

C.

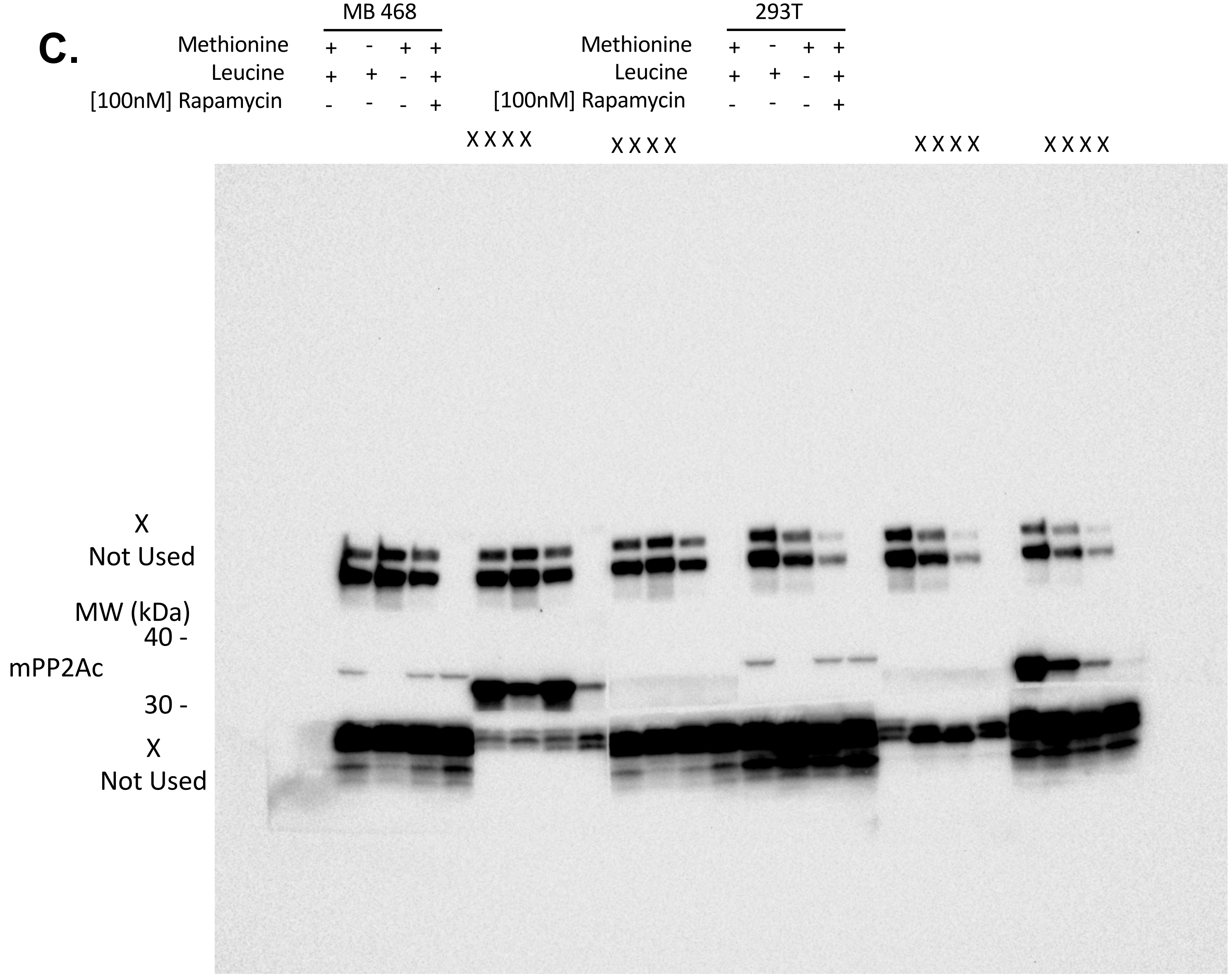
MB 468
293T
Methionine + - + +
Leucine + + - +
[100nM] Rapamycin - - - +
Methionine + - + +
Leucine + + - +
[100nM] Rapamycin - - - +
XXXX
XXXX
XXXX
XXXX
X
Not Used
MW (kDa)
40 -
mPP2Ac
30 -
X
Not Used

D.

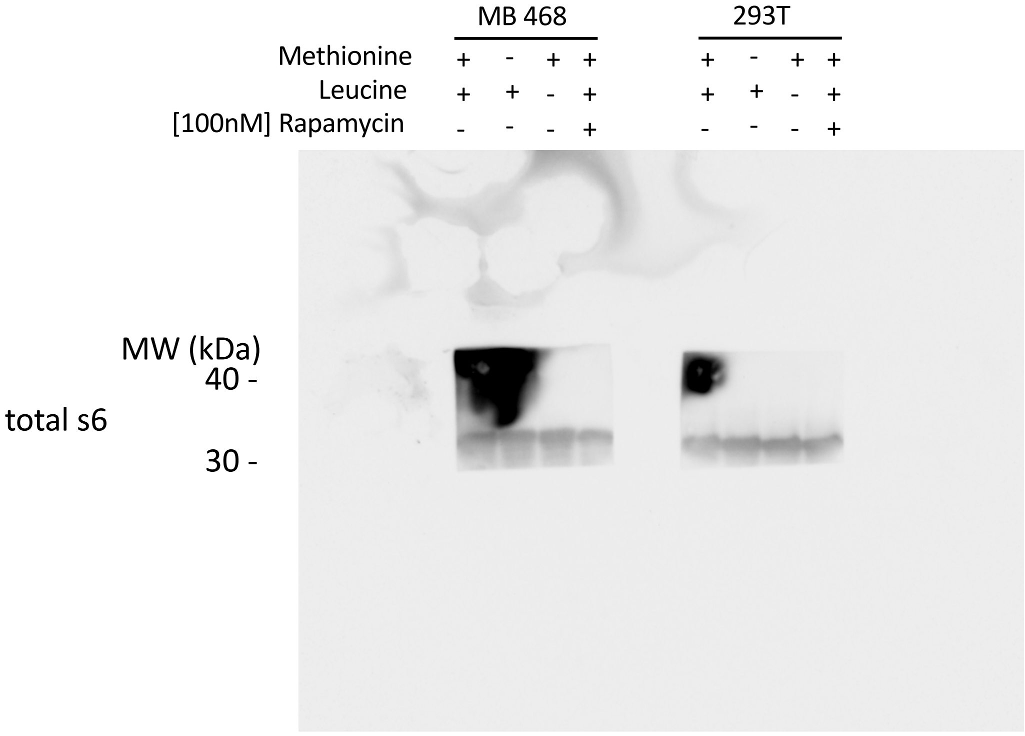
MB 468
293T
Methionine + - + +
Leucine + + - +
[100nM] Rapamycin - - - +
MW (kDa)
40 -
total s6
30 -

E.

Cell line		pS6	Total S6	Methyl PP2AC	Total PP2AC
MB468	**Complete Media**	7113898	774033	1299645	14762498
	**Methionine Depletion**	3512520	903201	35415	11659122
	**Leucine Depletion**	6433834	2864004	1318905	11681314
	**100nM Rapamycin**	464706	1807494	1762695	11292662
293T	**Complete Media**	7169778	2050893	2167560	11899146
	**Methionine Depletion**	2098294	2861820	51705	16345138
	**Leucine Depletion**	372174	3503292	1581840	12197862
	**100nM Rapamycin**	10274	2434731	1908180	12049526

Figure S18 : Uncropped blots relating to Figure 5A
A-D) Uncropped blots E) Densitometry (using total adjusted band volume from BioRad ImageLab)
Note: Membranes were cut to accommodate multiple antibodies

A.

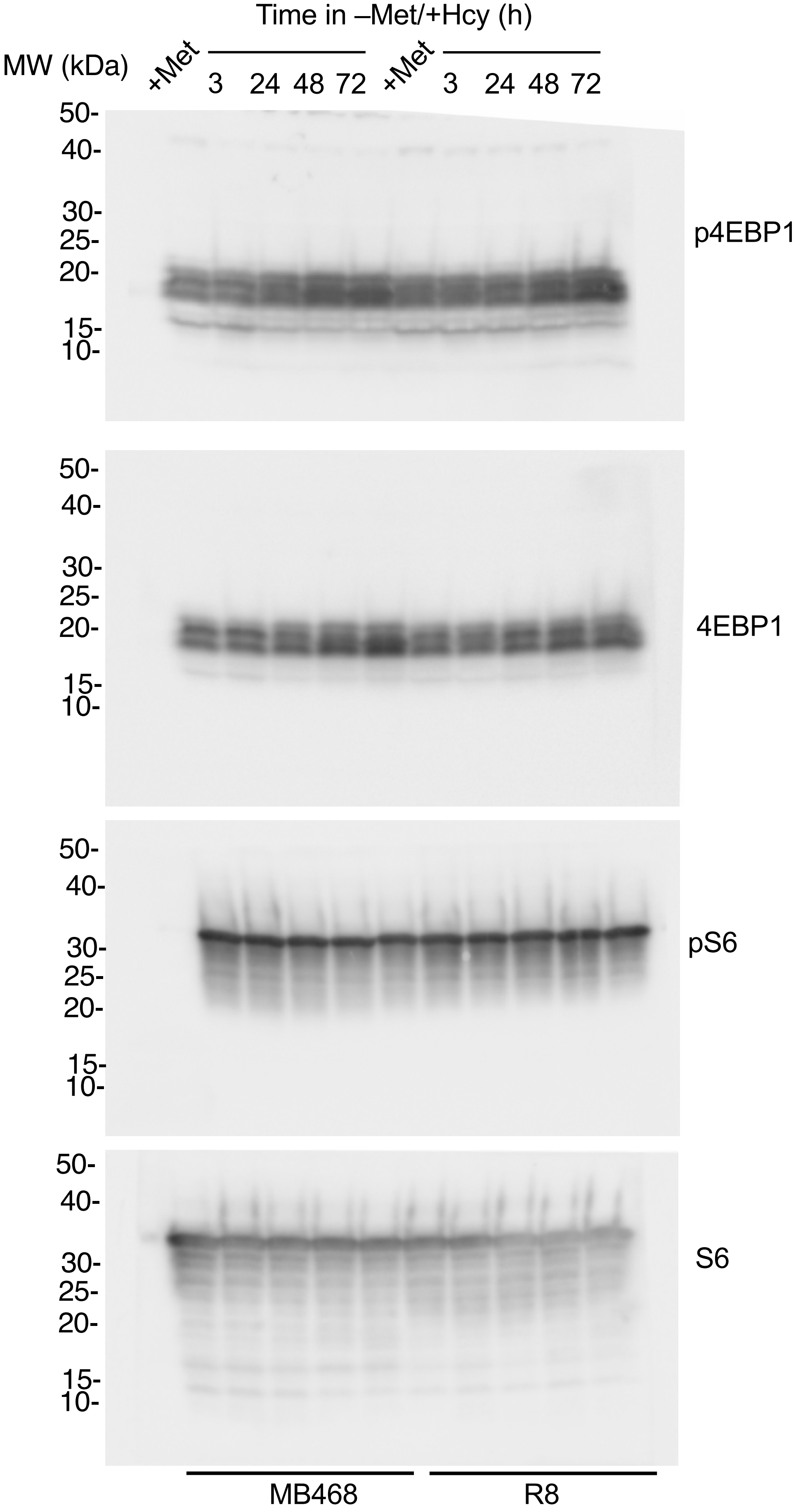
Time in –Met/+Hcy (h)
MW (kDa)
+Met
3
24
48
72
+Met
3
24
48
72
50-
40-
30-
25-
20-
15-
10-
p4EBP1
4EBP1
pS6
S6
MB468
R8

B.

	MB468					R8				
	Met	3	24	48	72	Met	3	24	48	72
p4EBP1	23979096	28208328	85180083	35490420	37045017	22218843	84256599	25246488	25805523	25141285
4EBP1	10508349	11568735	10761960	54362034	58597347	9761373	43995975	10988415	10896996	58470048
pS6	46456260	48131300	39416608	51829090	42261310	41448975	48212798	51268085	57745960	59282525
S6	2576340	1779960	1830996	4289220	3544827	3247055	3256422	817812	666798	2212548

Figure S19 : Uncropped blots relating to Figure 5B

A) Uncropped blots B) Densitometry (using total adjusted band volume from BioRad ImageLab)

Note: Membranes were cut to accommodate multiple antibodies

A.

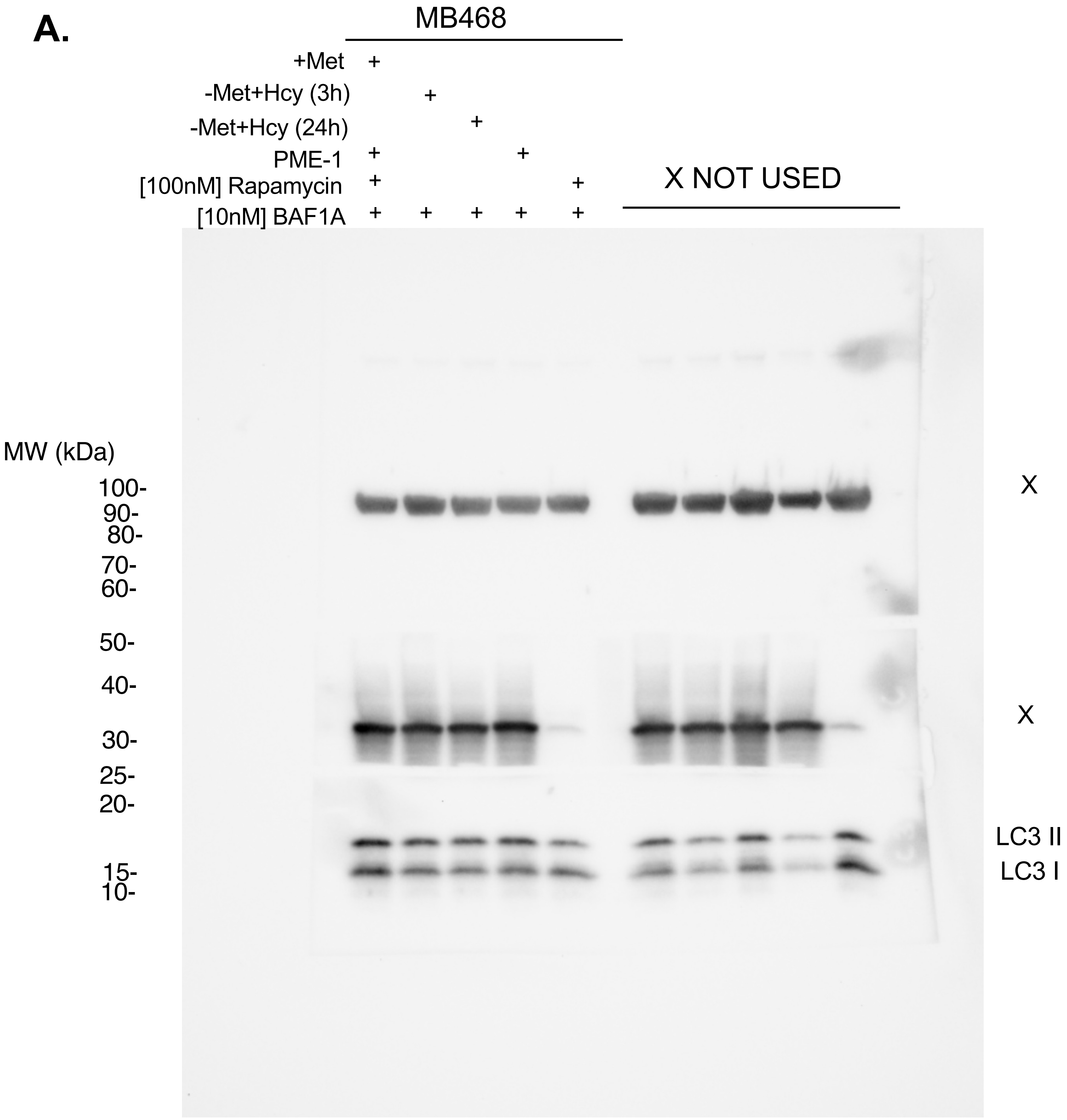
MB468
+Met
-Met+Hcy (3h)
-Met+Hcy (24h)
PME-1
[100nM] Rapamycin
[10nM] BAF1A
X NOT USED
MW (kDa)
100-
90-
80-
70-
60-
50-
40-
30-
25-
20-
15-
10-
X
X
LC3 II
LC3 I

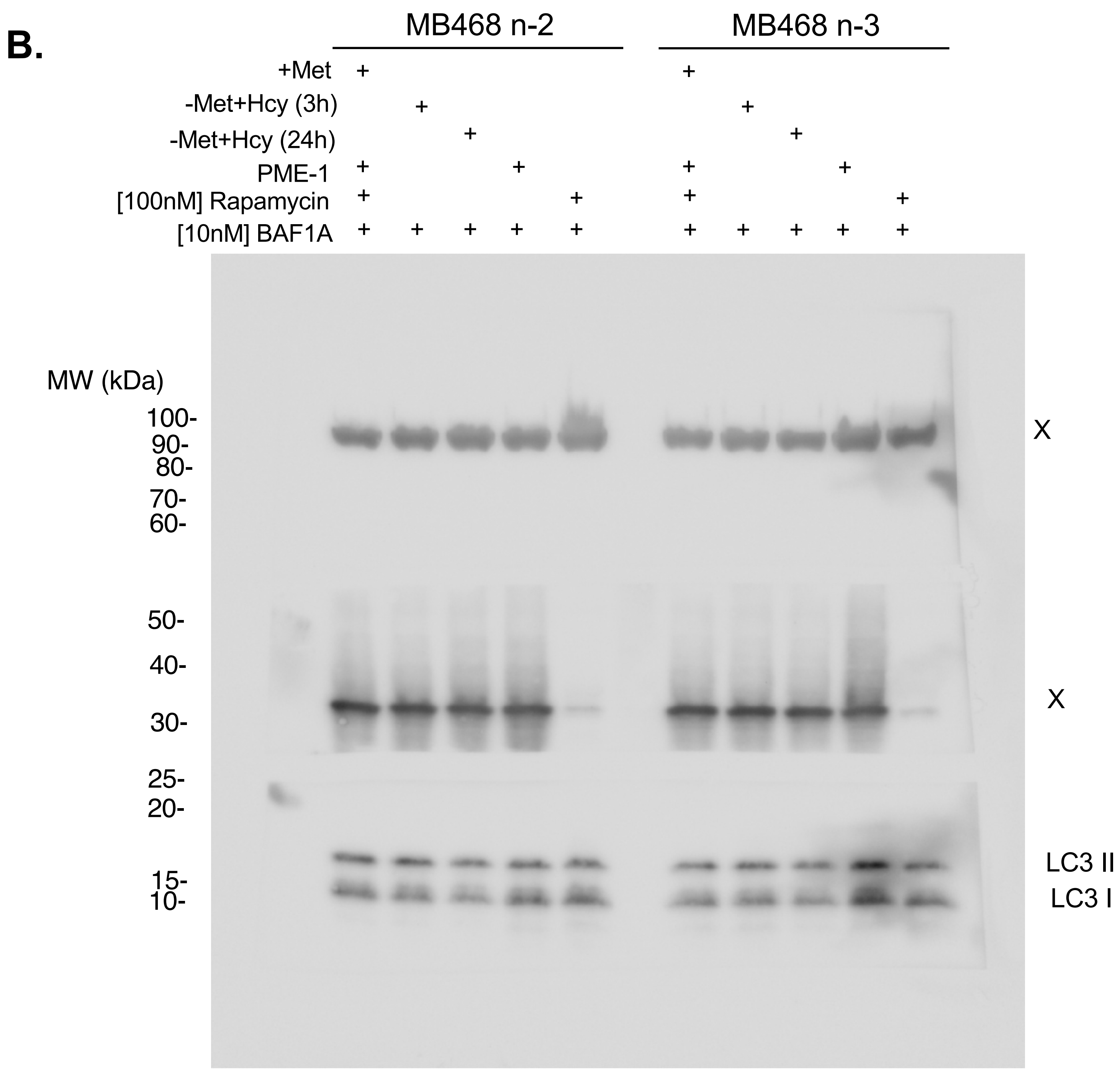

Replicates used for quantification

C.

		LC3 top band	LC bottom band	ratio
N-1	Met	7671093	9101718	1.18649559
	3h	5830192	7112359	1.21991849
	24h	5125507	6163841	1.20258172
	PME1	5949547	6960740	1.16996134
	Rapa	4257960	8265060	1.94108446
N-2	Met	6283523	7823148	1.24502576
	3h	5899189	6291916	1.06657305
	24h	4352697	4704331	1.08078532
	PME1	5579928	8536117	1.52978981
	Rapa	4924620	8637378	1.75391766
N-3	Met	4710285	6196660	1.31555946
	3h	5658575	6628370	1.17138502
	24h	4405420	4011315	0.91054088
	PME1	8646850	10625425	1.22882032
	Rapa	4867720	7388865	1.51793139

Figure S20 : Uncropped blots relating to Figure 5C
A and B) Uncropped blots C) Densitometry (using total adjusted band volume from BioRad ImageLab)
Note: Membranes were cut to accommodate multiple antibodies

A.

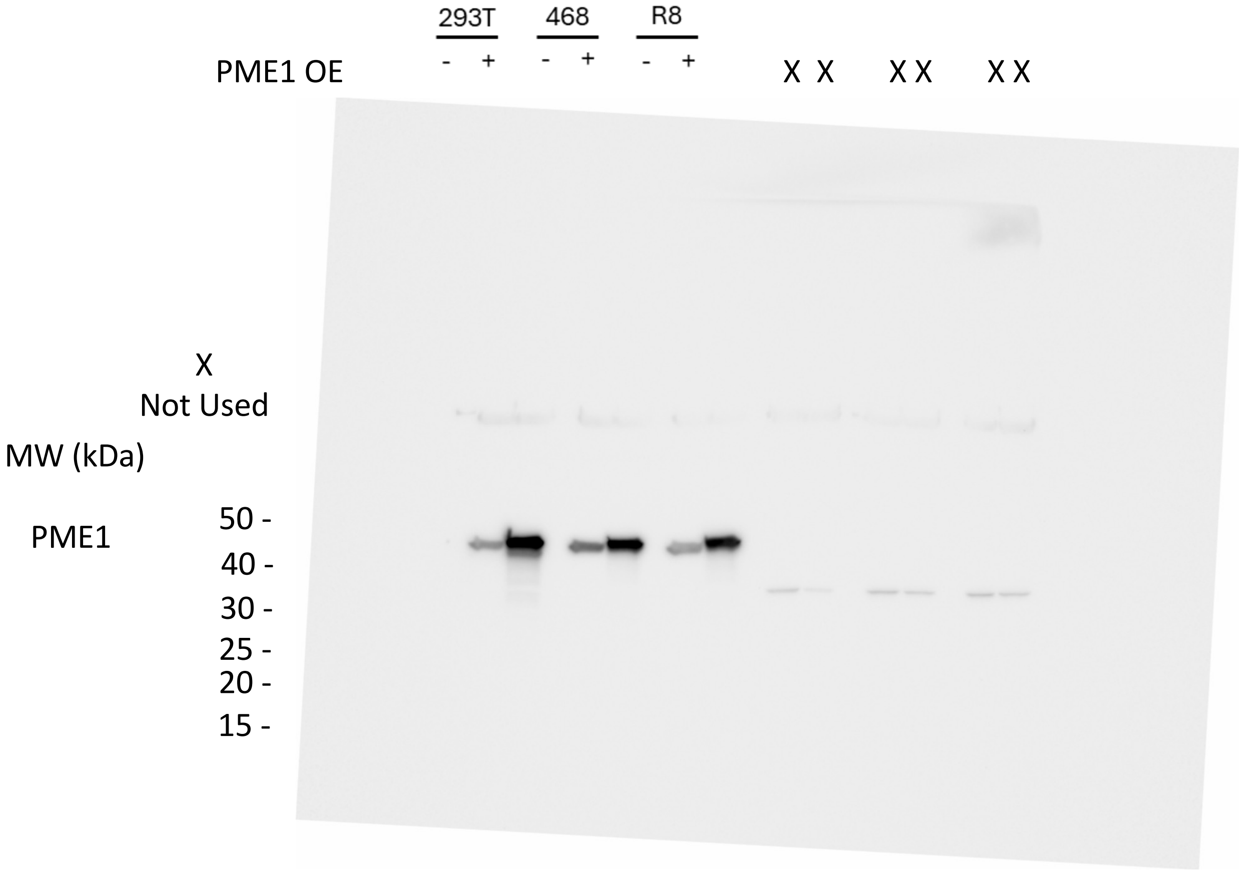
293T
468
R8
PME1 OE
- + - + - +
X X X X X X
X
Not Used
MW (kDa)
PME1
50 -
40 -
30 -
25 -
20 -
15 -

B.

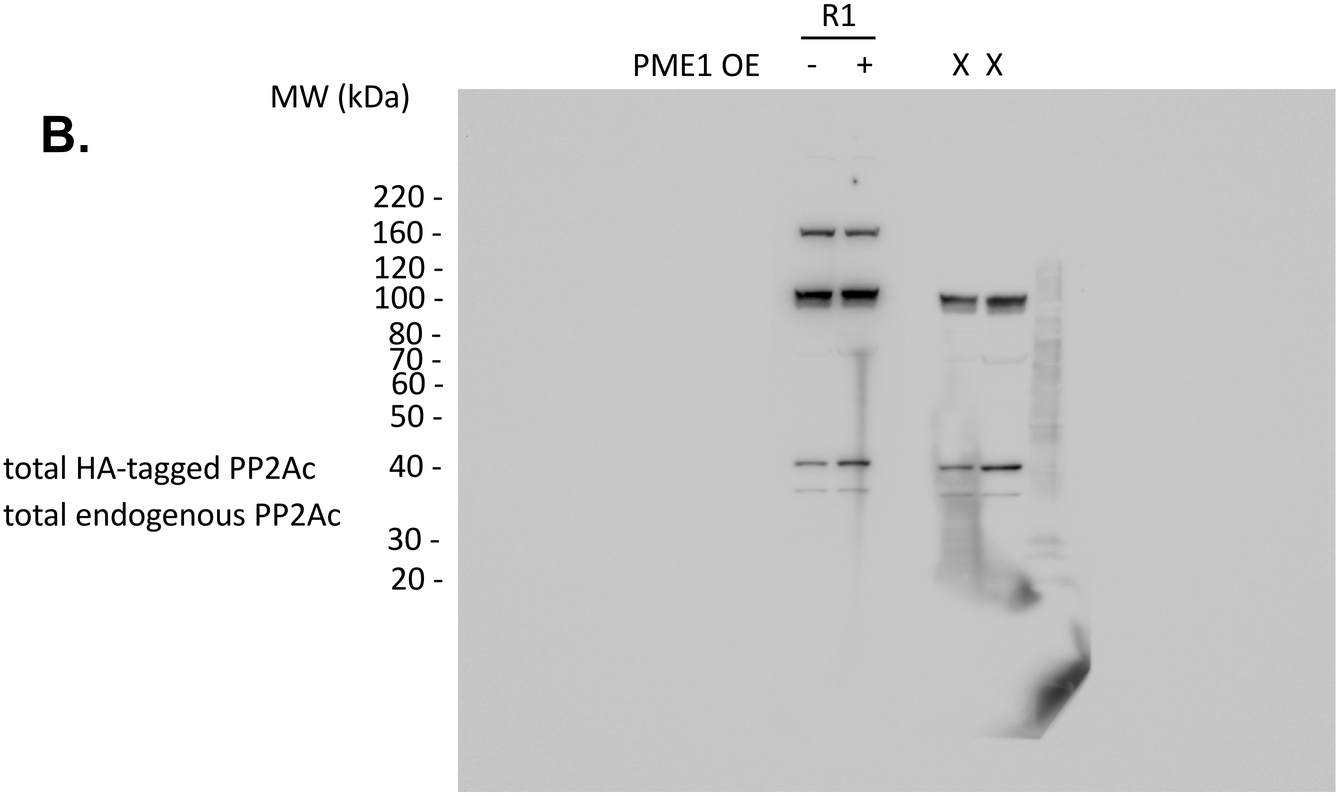
R1
PME1 OE
- +
X X
MW (kDa)
220 -
160 -
120 -
100 -
80 -
70 -
60 -
50 -
40 -
30 -
20 -
total HA-tagged PP2Ac
total endogenous PP2Ac

C.

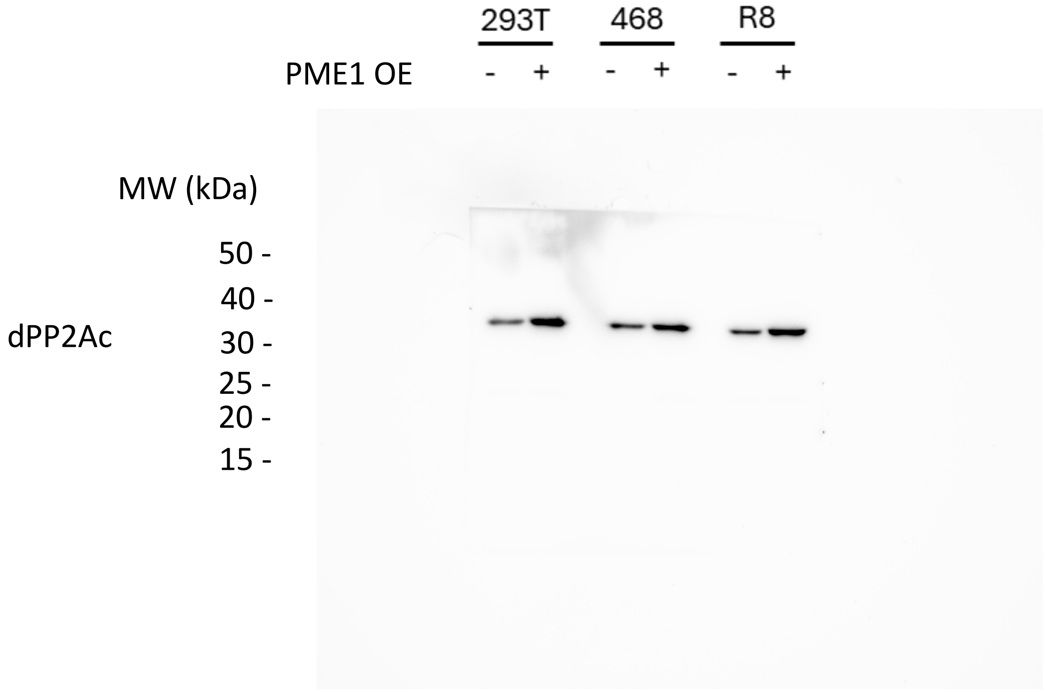
293T
468
R8
PME1 OE
- + - + - +
MW (kDa)
50 -
40 -
30 -
25 -
20 -
15 -
dPP2Ac

D.

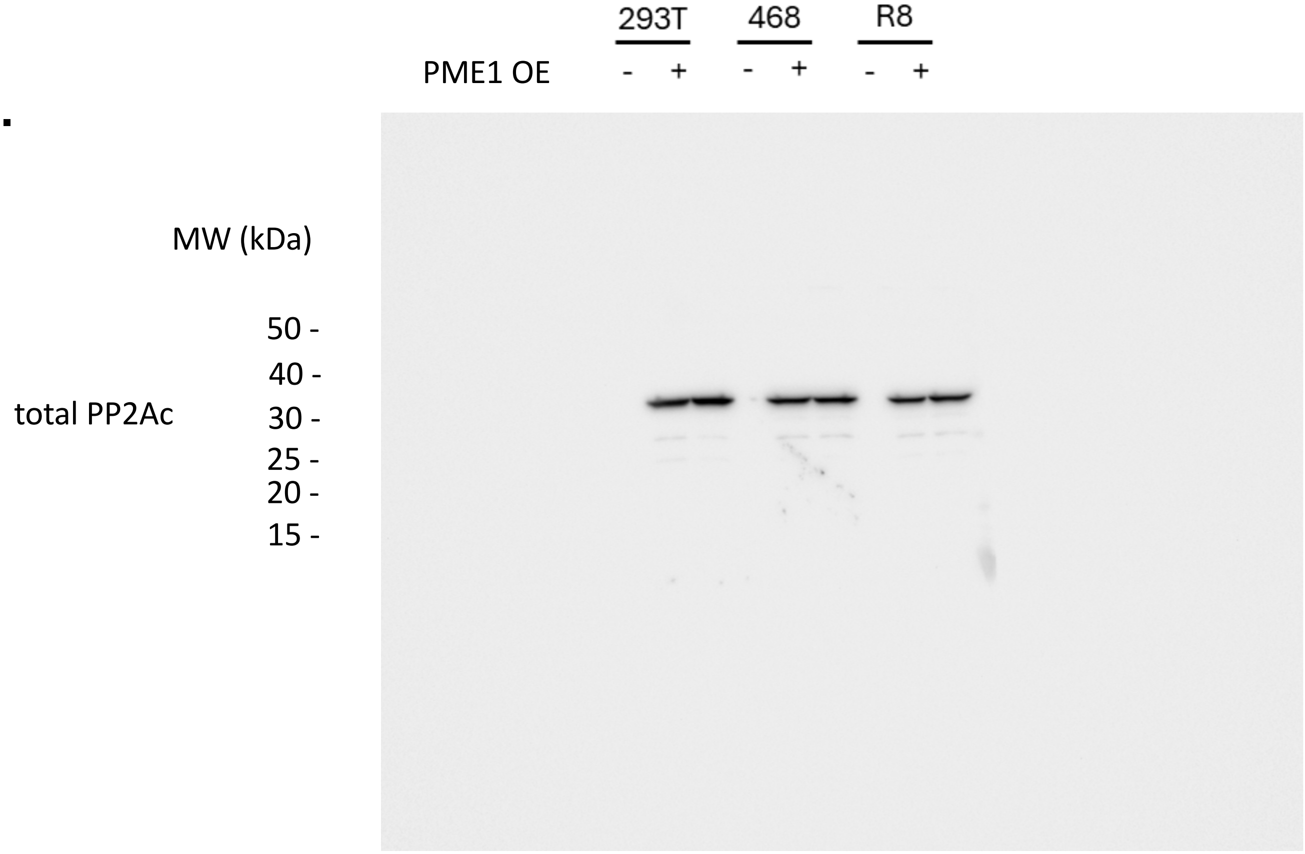
293T
468
R8
PME1 OE
- + - + - +
MW (kDa)
50 -
40 -
30 -
25 -
20 -
15 -
total PP2Ac

E.

	R1		R1	
PME1 OE	-	+	-	+

MW (kDa)

220 -
160 -
120 -
100 -
80 -
70 -
60 -
50 -
40 -
30 -
20 -

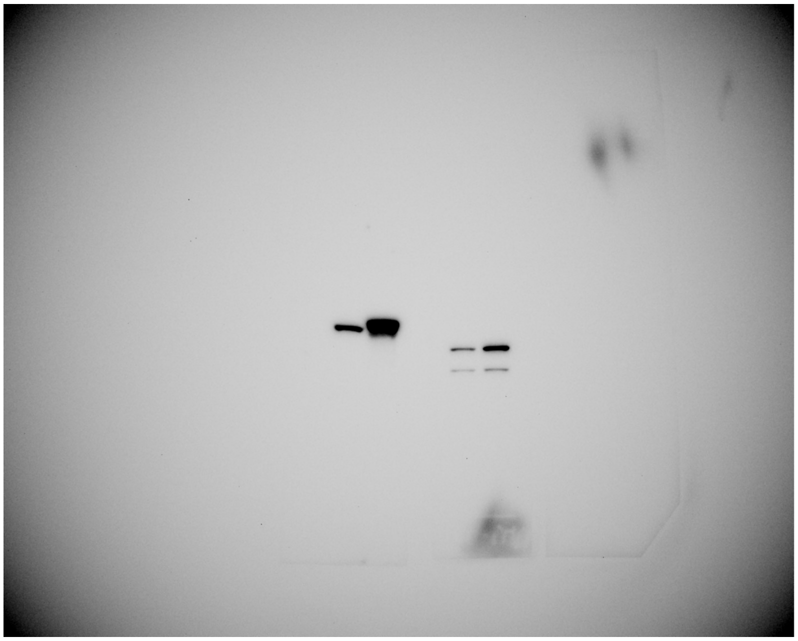

PME1
HA-tagged dPP2Ac
endogenous dPP2Ac

F.

	R1		R1			
PME1 OE	-	+	-	+	X	X

MW (kDa)

220 -
160 -
120 -
100 -
80 -
70 -
60 -
50 -
40 -
30 -
20 -

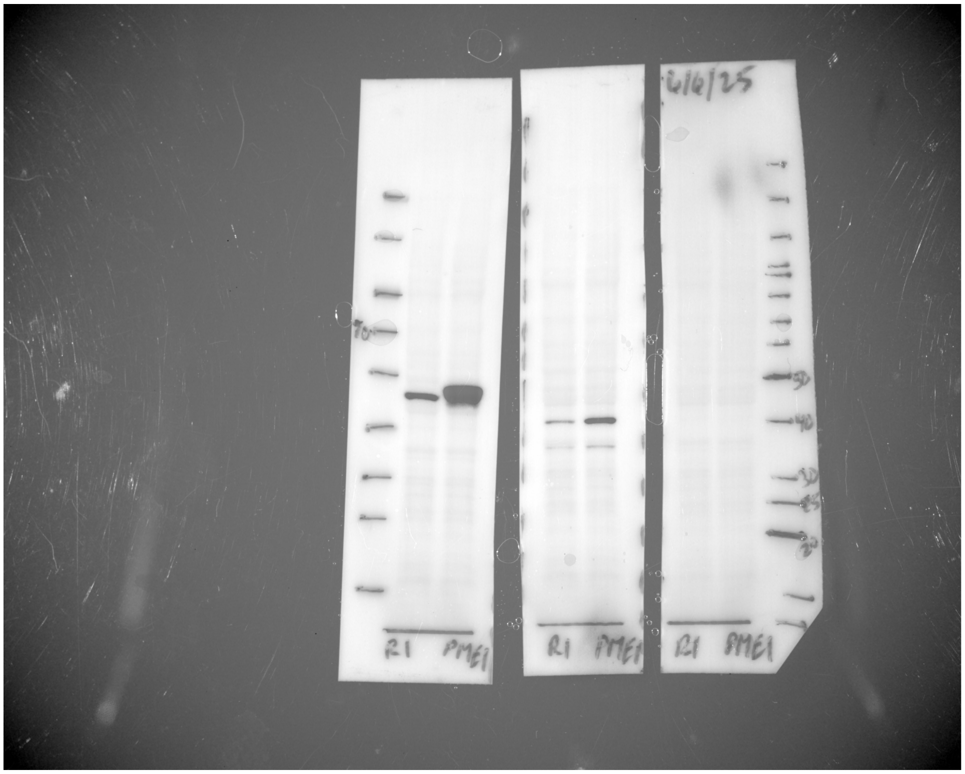

PME1

HA-tagged dPP2Ac

endogenous dPP2Ac

(Shown with overlay of cut membranes for clarity)

G.

	Total PP2AC	Demethylated PP2AC	PME1
**293T**	14041860	79923460	6539822
**293T PME1**	16812712	2.07E+08	39665740
**MB468**	13599660	93892190	8358406
**MB468 PME1**	14316962	1.59E+08	23986622
**R8**	11069539	77975940	5314764
**R8 PME1**	12042312	1.92E+08	19797778
**R1**	3563280	572000	1860875
**R1 PME1**	7795368	1916625	9890500

Figure S21 : Uncropped blots relating to Figure 6A and 6B A-F) Uncropped blots G) Densitometry (using total adjusted band volume from BioRad ImageLab)

Note: Membranes were cut to accommodate multiple antibodies

A.

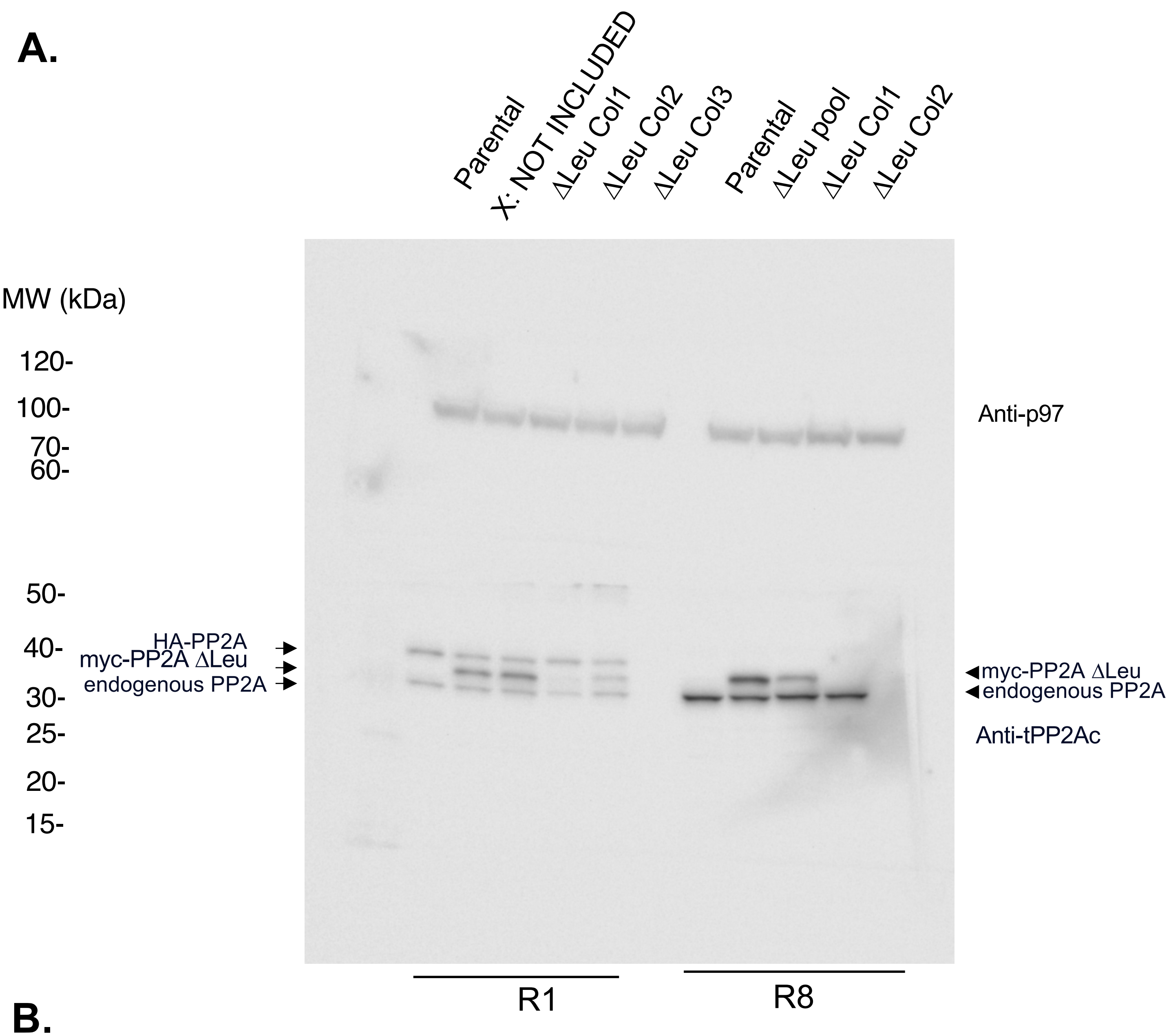

B.

	Parental	ΔLeu col1	ΔLeu col2	ΔLeu col3	Parental	ΔLeu pool	ΔLeu col1	ΔLeu col2
**tPP2A**	9010960	8121840	4031280	4320000	21693760	21094720	16931840	17262800
**p97**	9290585	8199610	7572990	7730155	6839865	6705990	8679435	9038726

Figure S22 : Uncropped blots relating to Figure 6C and 6D A) Uncropped blots B) Densitometry (using total adjusted band volume from BioRad ImageLab)

Note: Membranes were cut to accommodate multiple antibodies

A.

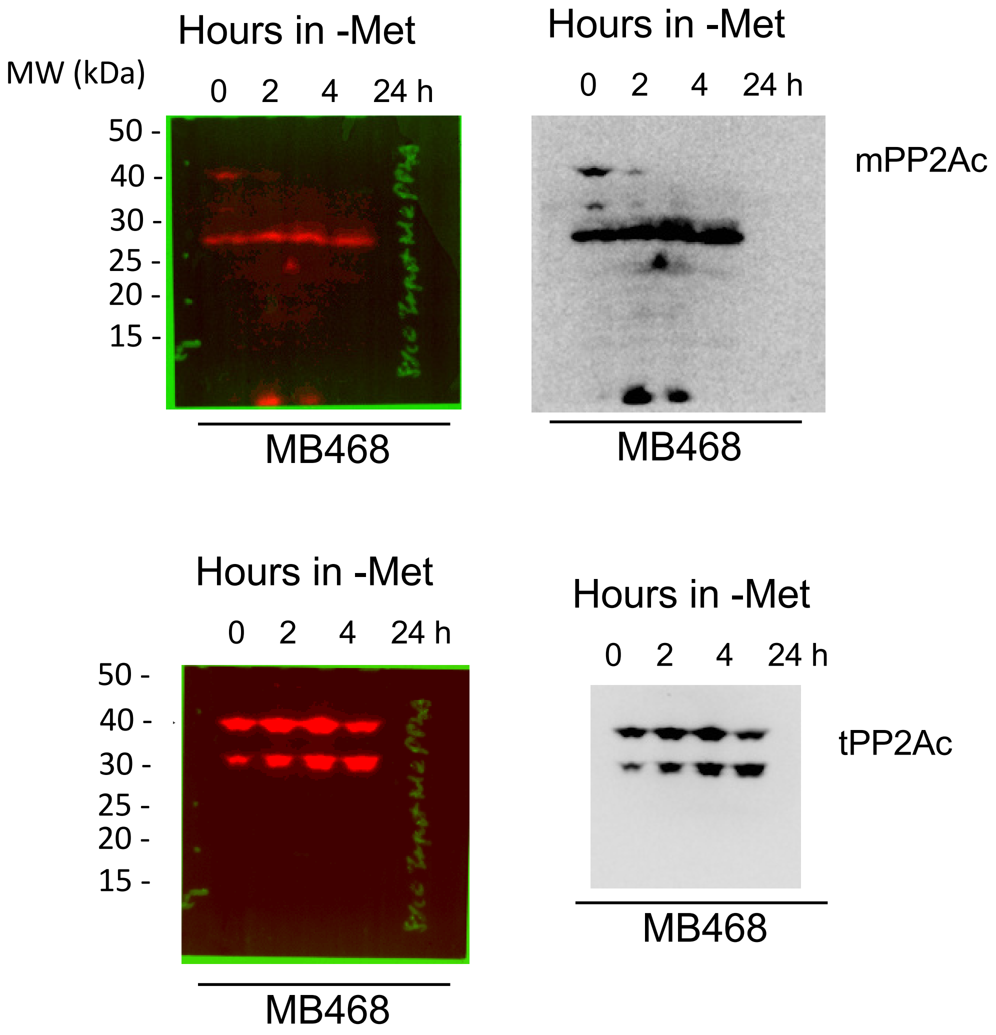

B.

	0	2	4	24
**mPP2A**	25994	5781	492	902
**tPP2A**	40443	51363	68133	53781

Figure S23 : Uncropped blots relating to Figure S11
A) Uncropped blots B) Densitometry (using total adjusted band volume from BioRad ImageLab)
Note: Membranes were cut to accommodate multiple antibodies
